# The Role of the Mammalian DNA End-processing Enzyme Polynucleotide Kinase 3’-Phosphatase in Spinocerebellar Ataxia Type 3 Pathogenesis

**DOI:** 10.1371/journal.pgen.1004749

**Published:** 2015-01-29

**Authors:** Arpita Chatterjee, Saikat Saha, Anirban Chakraborty, Anabela Silva-Fernandes, Santi M. Mandal, Andreia Neves-Carvalho, Yongping Liu, Raj K. Pandita, Muralidhar L. Hegde, Pavana M. Hegde, Istvan Boldogh, Tetsuo Ashizawa, Arnulf H. Koeppen, Tej K. Pandita, Patricia Maciel, Partha S. Sarkar, Tapas K. Hazra

**Affiliations:** 1 Department of Internal Medicine, University of Texas Medical Branch, Galveston, Texas, United States of America; 2 School of Health Sciences, Life and Health Sciences Research Institute (ICVS), University of Minho, Braga, Portugal; 3 ICVS/3B’s—PT Government Associate Laboratory, Braga/Guimarães, Portugal; 4 Department of Neurology and Neuroscience and Cell Biology, University of Texas Medical Branch, Galveston, Texas, United States of America; 5 Department of Radiation Oncology, University of Texas Southwestern Medical Center, Dallas, Texas, United States of America; 6 Department of Radiation Oncology, The Houston Methodist Research Institute, Houston, Texas, United States of America; 7 Department of Biochemistry & Molecular Biology, University of Texas Medical Branch, Galveston, Texas, United States of America; 8 Department of Microbiology & Immunology; University of Texas Medical Branch, Galveston, Texas, United States of America; 9 Department of Neurology, University of Florida, Gainesville, Florida, United States of America; 10 Department of Neurology, Albany Stratton VA Medical Center, Albany, New York, United States of America; The Hospital for Sick Children and University of Toronto, Canada

## Abstract

DNA strand-breaks (SBs) with non-ligatable ends are generated by ionizing radiation, oxidative stress, various chemotherapeutic agents, and also as base excision repair (BER) intermediates. Several neurological diseases have already been identified as being due to a deficiency in DNA end-processing activities. Two common dirty ends, 3’-P and 5’-OH, are processed by mammalian polynucleotide kinase 3’-phosphatase (PNKP), a bifunctional enzyme with 3’-phosphatase and 5’-kinase activities. We have made the unexpected observation that PNKP stably associates with Ataxin-3 (ATXN3), a polyglutamine repeat-containing protein mutated in spinocerebellar ataxia type 3 (SCA3), also known as Machado-Joseph Disease (MJD). This disease is one of the most common dominantly inherited ataxias worldwide; the defect in SCA3 is due to CAG repeat expansion (from the normal 14–41 to 55–82 repeats) in the ATXN3 coding region. However, how the expanded form gains its toxic function is still not clearly understood. Here we report that purified wild-type (WT) ATXN3 stimulates, and by contrast the mutant form specifically inhibits, PNKP’s 3’ phosphatase activity *in vitro*. ATXN3-deficient cells also show decreased PNKP activity. Furthermore, transgenic mice conditionally expressing the pathological form of human ATXN3 also showed decreased 3’-phosphatase activity of PNKP, mostly in the deep cerebellar nuclei, one of the most affected regions in MJD patients’ brain. Finally, long amplicon quantitative PCR analysis of human MJD patients’ brain samples showed a significant accumulation of DNA strand breaks. Our results thus indicate that the accumulation of DNA strand breaks due to functional deficiency of PNKP is etiologically linked to the pathogenesis of SCA3/MJD.

## Author Summary

We report that human polynucleotide kinase 3’-phosphatase (PNKP), a major DNA strand break repair enzyme, stably associates with Ataxin-3 (ATXN3). This protein contains repeats of the amino acid glutamine, and the expansion of these repeats from 14–41 to 55–82 glutamines leads to a neurological disorder called Spinocerebellar ataxia type 3 (SCA3), also known as Machado-Joseph Disease (MJD). However, how this expansion of glutamine leads to ataxia has remained unclear. Here we show that normal ATXN3 protein stimulates, but the expanded ATXN3 inhibits, PNKP’s DNA repair activity, causing an accumulation of DNA damage. Furthermore, a SCA3 mouse model showed decreased PNKP activity, mostly in a region that is highly affected in MJD patients’ brains. Analysis of human MJD patients’ neuronal DNA showed significant accumulation of DNA strand breaks. Collectively, the accumulation of DNA damage due to decreased PNKP repair activity is likely to induce neuronal cell death, a hallmark of SCA3/MJD pathogenesis.

## Introduction

DNA strand breaks (SBs), both single-stranded (SSBs) and double-stranded (DSBs), with various “dirty” DNA ends are among the most toxic and mutagenic lesions in mammalian genomes, because such ends block the action of DNA polymerases and DNA ligases; the conventional 3’-OH (hydroxyl) and 5’-P (phosphate) ends must be restored for gap-filling and DNA ligation to occur during repair for maintaining genomic integrity. The end-processing steps have now become a major focus because of the observation that two of the proteins involved in this process (Aprataxin and TDP1) are mutated in hereditary neurodegenerative diseases [1–4]. Human polynucleotide kinase 3’-phosphatase (PNKP) is another DNA end-processing enzyme; it removes the 3’-P group [5–7] and catalyzes the phosphorylation of 5’-OH termini [(generated by some nucleases, and as intermediates of topoisomerase cleavage [5,8], and thus is involved in the repair of both SSBs via the SSB repair (SSBR) pathway and DSBs via non-homologous end joining. It has recently been reported that mutation in PNKP or its reduced level causes an autosomal recessive disease (termed MCSZ); characterized by microcephaly, intractable seizures, and developmental delay [9]. Another very recent report showed cerebellar atrophy and polyneuropathy in humans due to PNKP mutation [10]. Unrepaired SBs can impact cell fate in several ways. The most likely effect in proliferating cells is the blockage or collapse of DNA replication forks during the S phase. However, replicating cells have the ability to repair such DSBs in the nuclear genome via homologous recombination [11,12]. In non-proliferating cells, such as postmitotic neurons, SBs might cause stalling of RNA polymerase II during transcription, particularly at the 3’-P ends, and induce cell death via p53-mediated activation of apoptotic pathways [13]. Hence, SBs pose a significant threat to maintaining genomic integrity and neuronal cell survival [14].

While screening for PNKP-associated proteins, we identified Ataxin-3 (ATXN3) in the PNKP immunopull-down complex. Eukaryotic ATXN3 is ubiquitously expressed in various tissues and also found both in the cytoplasm and nucleus of neuronal cells [15–18]; its normal biological function, despite many serious efforts, is not yet fully understood. It has been suggested that ATXN3 is a multifunctional protein that plays a role in transcriptional regulation, ubiquitination and protein homeostasis maintenance via its deubiquitinating activity [19–22]. ATXN3 is a polyglutamine (polyQ)-containing protein that carries 14–41 polyQ repeats in the normal population; when the polyQ length expands beyond 60, it causes Spinocerebellar ataxia type 3 (SCA3; OMIM:109150), also known as Machado-Joseph Disease (MJD), a hereditary neurodegenerative disorder characterized by gait ataxia, dysarthria and ophthalmoplegia, variably associated with spasticity, dystonia or amyotrophy and peripheral neuropathy [23]. Although it is a rare disease, genetic testing of large cohorts of ataxia patients identified MJD as one of the most commonly inherited ataxias worldwide [24,25]. It has been widely assumed that protein misfolding and aggregation is a major mechanism of SCA3 pathogenesis, but a clear consensus regarding the molecular mechanism of the toxic gain of function of the pathological form of ATXN3 has still not been reached. Here we report that WT ATXN3 stimulates, and by contrast mutant ATXN3 blocks the DNA 3’-end-processing activity of PNKP, and the resulting accumulation of DNA SBs may contribute significantly to SCA3 pathogenesis via modulating the DNA damage-response pathway.

## Results

### Identification of Ataxin-3 in the PNKP complex

**Figure 1:**
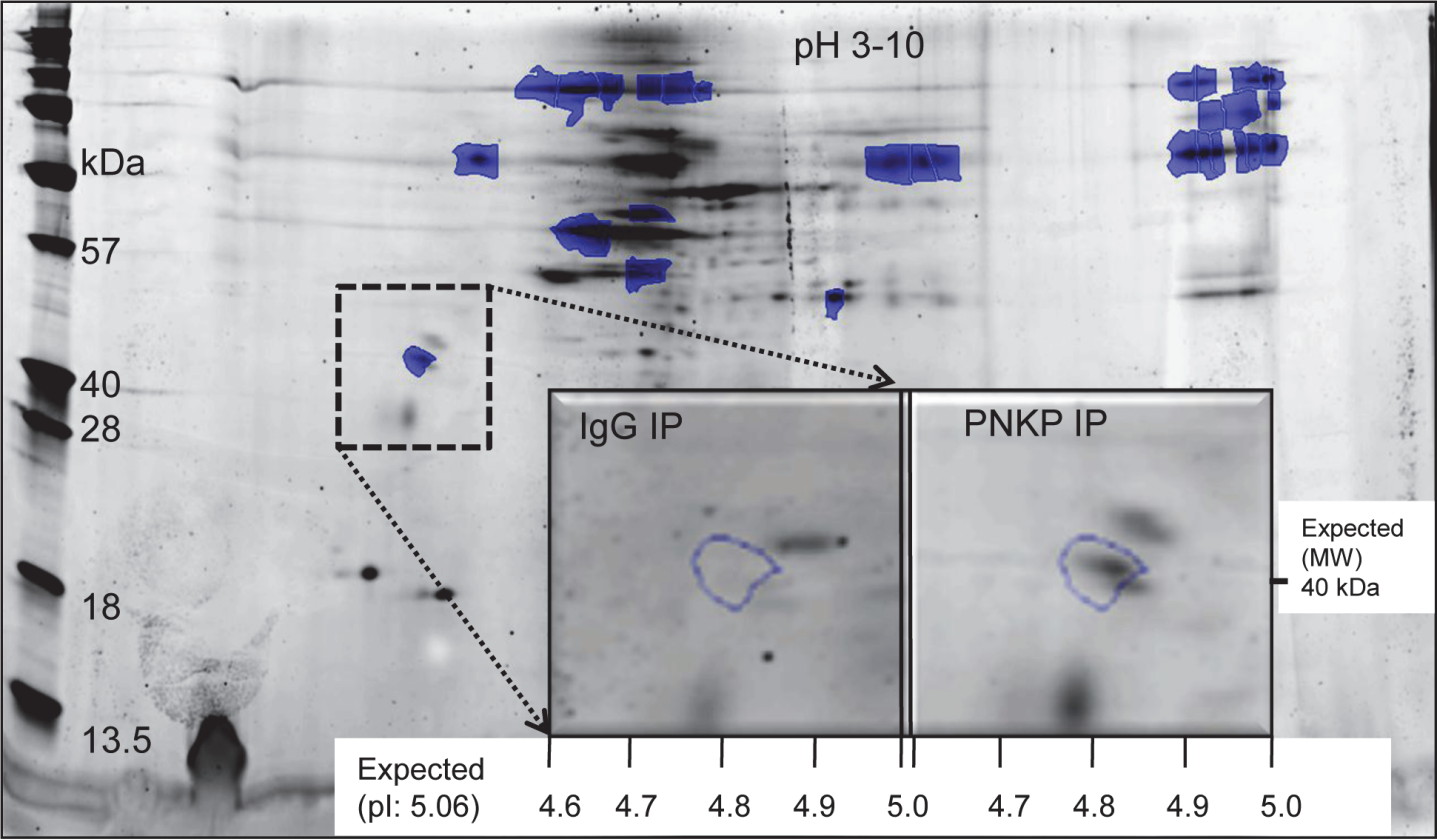
Identification of ATXN3 in the PNKP IP by 2D gel and MALDI-TOF-TOF MS analysis. The spot annotated within the dashed box (absent in the control IP) was identified as human ATXN3 by subsequent MS/MS analysis (S1 Fig.).

We and several other groups have reconstituted PNKP-mediated DNA strand-break repair *in vitro*, and also characterized the individual steps in the multistep repair process [26,27]. However, the *in vivo* repair process is far more complicated; for efficient DNA damage processing and repair, most, if not all of the components involved in a particular repair pathway, form complex(es) in a dynamic fashion within a “repair factory” [28]. To delineate the different steps and molecular mechanisms of the PNKP-mediated repair process, we screened for PNKP’s interacting partners via 2D-gel electrophoresis and subsequent mass spectroscopic (MALDI-TOF-TOF MS) analysis of a large-scale affinity pull-down of the PNKP immunocomplex. In the 500 mM (most tightly bound) salt eluate from the PNKP complex, we identified ATXN3 (Figs. 1 and S1), a poly-glutamine-containing protein with no known role in DNA repair, except for its association with HHR23 proteins, which are involved in nucleotide excision repair [29]. Characterization of other pulled down proteins and their role in PNKP-mediated DNA strand-break repair are currently under investigation.

**Figure 2:**
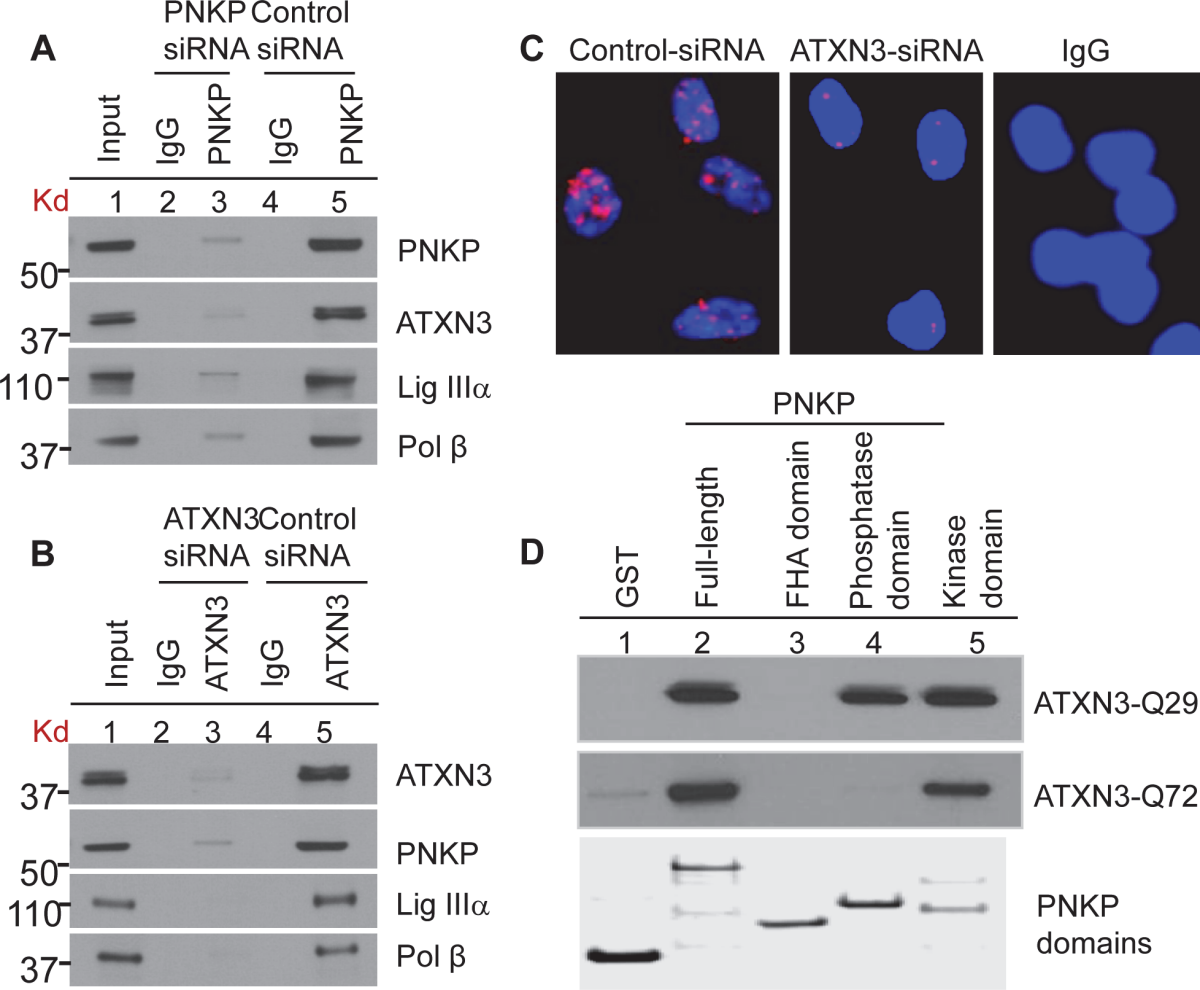
Characterization of the (A) PNKP and (B) ATXN3 immunocomplexes by Western blot analysis. HEK-293 cells were transfected with either PNKP siRNA or ATXN3 siRNA or control siRNA and the nuclear extracts (1 mg) prepared from those cells were IP’d with anti-PNKP Ab (BioBharati Life Science Pvt. Ltd, Kolkata, India; **2A**) or anti-ATXN3 Ab (Proteintech, **2B**) or with IgG as a control, and tested for the presence of PNKP- and ATXN3-associated proteins with Abs to the proteins shown on the right. **(C)** Detection of PNKP’s interaction with ATXN3 in SH-SY5Y cells by proximity ligation assays using a Duolink kit (Olink Bioscience, Uppsala, Sweden). Nuclei were counterstained with DAPI (blue). ATXN3-depleted (by siRNA) cells were used as a control to show the specificity of the interaction (middle panel). Right panel, Non-specific Ab (IgG) control **(D)** GST-PNKP pull-down of ATXN3 (WT and mutant) using purified GST-tagged full-length or three domains (FHA-, Kinase- and Phosphatase-domain) of PNKP, probed with anti-ATXN3. Bottom panel, Coomassie-stained gel of the corresponding PNKP domains, a second gel run in parallel.

To examine ATXN3’s association with PNKP, we immunoprecipitated (IP’d) PNKP and ATXN3 separately from the nuclear extract (NE, benzonase treated to remove DNA and RNA to avoid DNA-mediated co-immunoprecipitation) of human HEK-293 (human embryonic kidney cell line) (Figs. 2A and B) and SH-SY5Y (a human neuroblastoma cell line) cells (S2A and S2B Figs.) using the respective anti-protein (PNKP or ATXN3) antibody (Ab). The experiment was conducted in two cell lines to test the global nature of ATXN3’s interaction with PNKP and related repair proteins, and thus to confirm its general role in DNA SB repair. We confirmed the presence of ATXN3 in the PNKP IP, along with Polβ and Lig IIIα, the known PNKP-associated proteins (Figs. 2A and S2A) [30]. Moreover, the reverse IP with an anti-ATXN3 Ab showed the presence of PNKP, Polβ and Lig IIIα (Figs. 2B and S2B), suggesting that ATXN3 is indeed a part of the complex and plays a role in PNKP-mediated DNA SB repair. To test the specificity of the association between PNKP and ATXN3, we depleted PNKP (S3 Fig.) and ATXN3 (S4 Fig.) individually, using siRNAs. Immunoblot analysis of the whole gel shows a single band of PNKP (S3 Fig., ln 6) or ATXN3 (S4 Fig., ln 5) in the NE from control siRNA-treated cells that runs with the corresponding purified recombinant protein (used as marker). Significant depletion (∼85%) of the corresponding band (S3 Fig., ln 7 and S4 Fig., ln 6) was noted in the depleted extract. Importantly, IPs using the corresponding Ab (Fig. 2A and 2B, ln 3) clearly shows that depletion of PNKP or ATXN3 strongly decreases the levels of their partners in the complex (compare lane 5), indicating the specificities of both the Abs and the association of the proteins in the complex.

To further confirm the in-cell association of PNKP with ATXN3, we performed an *in situ* proximity ligation assay (PLA), in which the close physical association of two proteins is visualized by a fluorescent signal [31–33]. To assess the specificity of their interaction, cells were treated with control or ATXN3 siRNA; forty-eight hours after siRNA transfection, the cells were fixed, co-immunostained with PNKP (anti-mouse) and ATXN3 (anti-rabbit) Abs and performed PLA per the manufacturer’s protocol (Olink Bioscience). We randomly selected 50 cells and manually counted the numbers of PLA foci. It was found that control siRNA-treated cells had 10–12 PLA signals/cell whereas ATXN3 siRNA-treated cells had only 1–2 foci (Fig 2C). In addition, to assess the background levels of non-specific staining, cells were processed in the absence of antibodies; no fluorescence signals were detected, as was the case when control Abs were used in place of specific primary Abs (Fig 2C, right panel), further indicating specificities of PNKP’s association with ATXN3.

As a follow-up to our initial observation of ATXN3’s association with PNKP, we performed a GST pulldown using purified proteins to test the binary interaction of ATXN3-Q29 (WT with 29 polyQ repeats) and -Q72 (the pathological form with 72 polyQ repeats) with GST-PNKP. Binary interactions are often driven by the interaction of specific regions in the constituent proteins. PNKP has 3 domains: forkhead-associated domain (FHA; residues 1–119), phosphatase domain (P; residues 120–339) and kinase domain (K; residues 340–521). We thus separately expressed and purified GST-tagged full-length PNKP and all 3 of its domains from *E. coli*; equimolar solutions of each were then incubated separately with WT or mutant ATXN3 at 4°C for 4 h. Beads containing the interacting proteins were repeatedly washed with 200 mM salt-containing buffer. Fig. 2D shows that both forms of ATXN3 interacted directly with full-length as well as the kinase domain of PNKP. Importantly, the phosphatase domain interacted only with WT, but not with mutant ATXN3. We also performed far-Western analysis to detect binary interaction between full-length PNKP vs. WT or mutant ATXN3. S5 Fig. (top panel, lns 1& 2) shows that both forms of ATXN3 do interact with PNKP, but not with bovine serum albumin (BSA; ln 3, used as a control). All these data are also consistent with the findings in the accompanying manuscript by Gao et al. (Figs. 1, 2 and Figs. S1, S3 & S4) where it is shown, using various techniques, that both WT and mutant ATXN3 associate with PNKP in-cell, and in mouse and human brain tissues (control and SCA3).

### Wild-type ATXN3 promotes, whereas the mutant form inhibits, the 3’-phosphatase activity of PNKP *in vitro*

**Figure 3:**
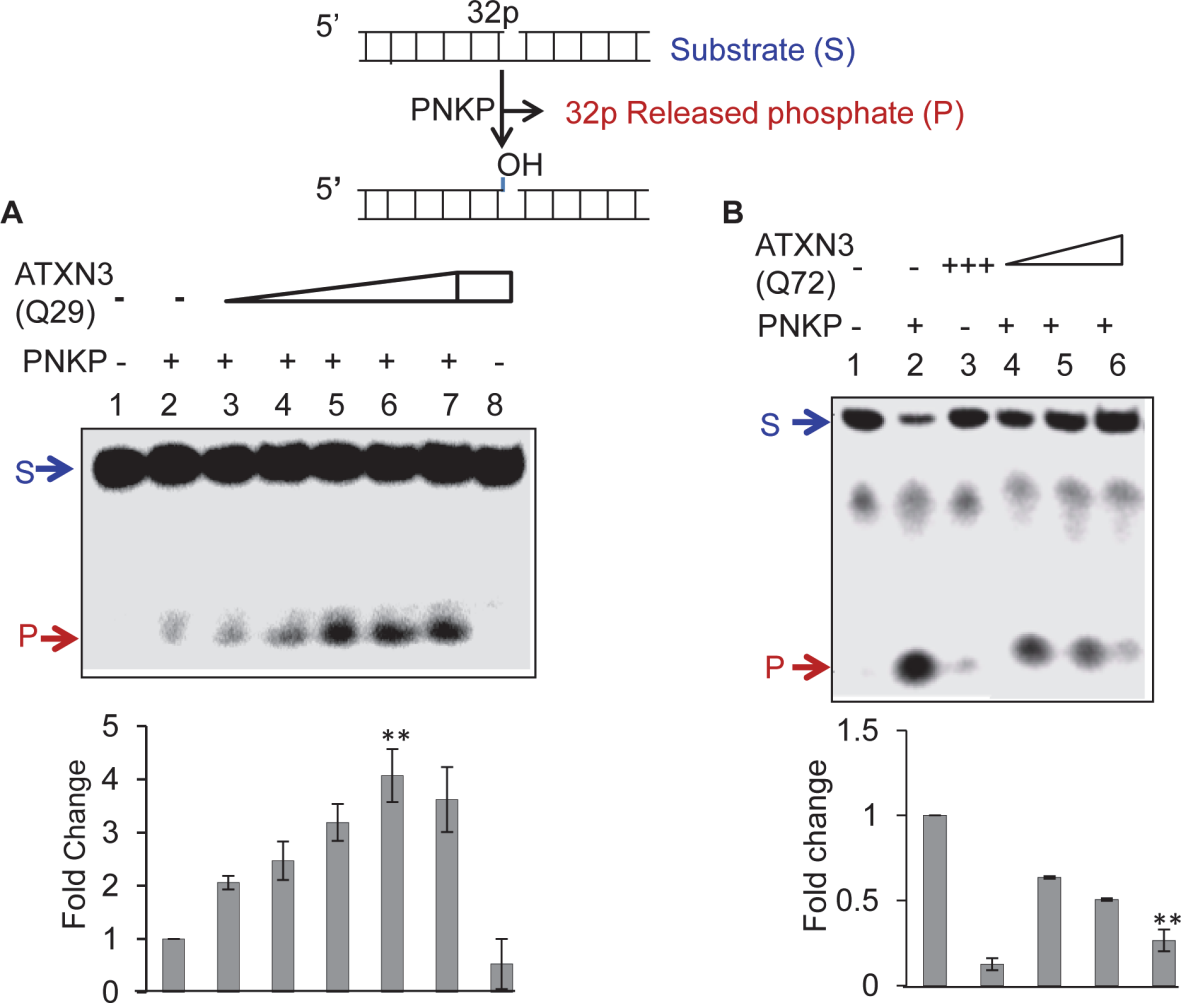
Effect of WT (Q29) or mutant (Q72) ATXN3 on PNKP’s 3’-phosphatase activity. **(A)** A ^32^P-labeled 3’-phosphate-containing oligo substrate (5 pmol) was incubated at 37°C for 10 min in buffer A (25 mM Tris-HCl, pH 7.5, 100 mM NaCl, 5 mM MgCl_2_, 1 mM DTT, 10% glycerol and 0.1 μg/μl acetylated BSA) with PNKP alone (50 fmol, ln 2) or ATXN3-Q29 alone (400 fmol, ln 8) or PNKP plus increasing amounts of Q29 (25, 50, 100, 200 and 400 fmol; lns 3–7). Ln 1, substrate only. Generation of the ^32^P-labelled 3’P-containing oligo substrate was described previously [53]. **(B)** Effect of mutant ATXN3 (Q72) on PNKP’s 3’-phosphatase activity. ^32^P-labelled 3’-phosphate-containing oligo substrate (2.5 pmol) was incubated with PNKP at 37°C for 20 min alone (50 fmol, ln 2), ATXN3-Q72 alone (200 fmol, ln 3), PNKP (50 fmol) plus increasing amounts of Q72 (50, 100 and 200 fmol, lns 4–6). Ln 1, no protein, substrate only. Quantitation of the products (released phosphate) is represented in the histogram (lower panel), with the activity of PNKP alone arbitrarily set as 1 (n = 3, ** = P< 0.01).

Given the stable interaction of PNKP with both forms of ATXN3, we tested the functional implications of these interactions by analyzing PNKP’s 3’-phosphatase activity. Fig. 3A shows that WT ATXN3 stimulated PNKP’s 3’-phosphatase activity (ln 2 vs. lns 3–7) ∼4-fold; in contrast, ATXN3-Q72 reproducibly and significantly abrogated PNKP’s activity (∼3.5-fold, Fig. 3B, ln 2 vs. ln 6; n = 3). Notably, neither the WT nor the mutant form of ATXN3 affected the activities of Polβ (S6A Fig., ln 1 vs. 2–3 and 4–5) or Lig IIIα (S6B Fig.), both of which associate with PNKP in a multiprotein complex that conducts SSBR [30]. These data indicate that the pathological form of ATXN3 specifically blocks PNKP’s 3’-phosphatase activity.

**Figure 4:**
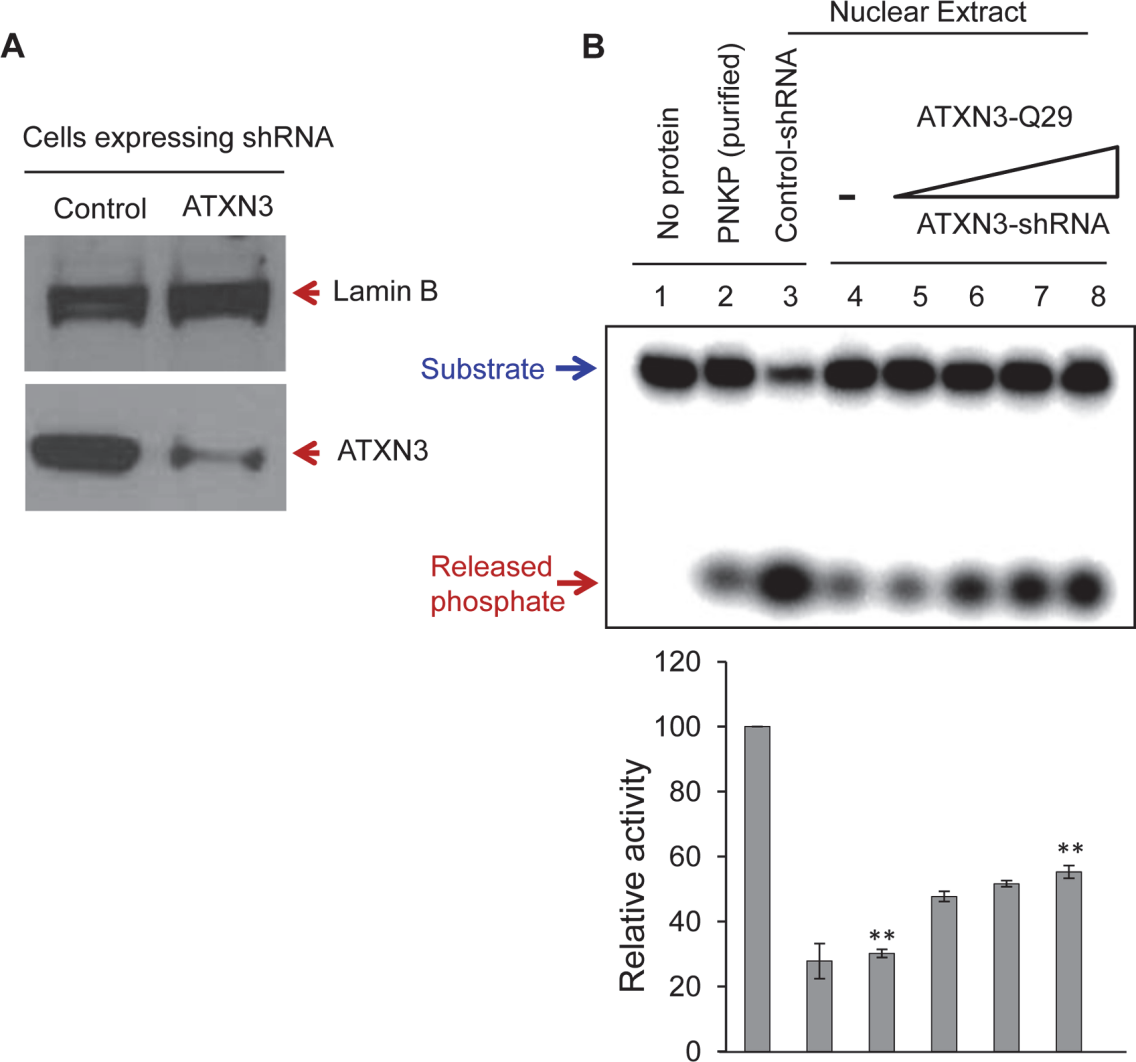
ATXN3 depletion decreases PNKP’s 3’-phosphatase activity. **(A)** Western blot analysis showing the level of expression of ATXN3 in SH-SY5Y cells stably expressing control vs. ATXN3-shRNA. Lamin B was used as a loading control. **(B)** A representative gel **(n = 3)** showing the 3’-phosphatase activity in the nuclear extract (200 ng) of control (ln 3) and ATXN3-depleted cells (ln 4). Lns 5–8, increasing amounts (10, 25, 50 and 100 fmol) of purified ATXN3 (WT) were added back to ATXN3-depleted nuclear extract. Quantitation of the products is represented in the histogram (n = 3, ** = P< 0.01).

To further examine the in-cell effects of ATXN3 on PNKP, we first examined the phosphatase activity in the nuclear extract of control vs. PNKP siRNA-treated cells. We found that PNKP depletion almost completely abrogated phosphate release, indicating that PNKP is the major, if not the only, 3’-phosphatase in mammalian cells (S7 Fig., ln 1 vs. ln 2). We then measured the phosphatase activity in the NE prepared from ATXN3-depleted (by ∼80%, Fig. 4A) vs. control shRNA-expressing cells. Fig. 4B shows a significant decrease in the phosphatase activity in ATXN3-depleted compared to control shRNA-expressing cells (by ∼70%, Fig. 4B, ln 3 vs. ln 4). Furthermore, addition of purified WT ATXN3 to the NE of ATXN3^shRNA^-depleted (Fig. 4B, lns 5–8) but not to PNKP-depleted (S7 Fig., ln 3) cells led to the recovery of phosphatase activity, thus confirming that the stimulation of phosphatase activity by ATXN3 in NE is PNKP-mediated. These results also confirmed ATXN3’s role in PNKP-mediated DNA SB repair.

### ATXN3 depletion causes DNA strand-break accumulation and delayed repair of oxidative stress-induced DNA strand breaks

**Figure 5:**
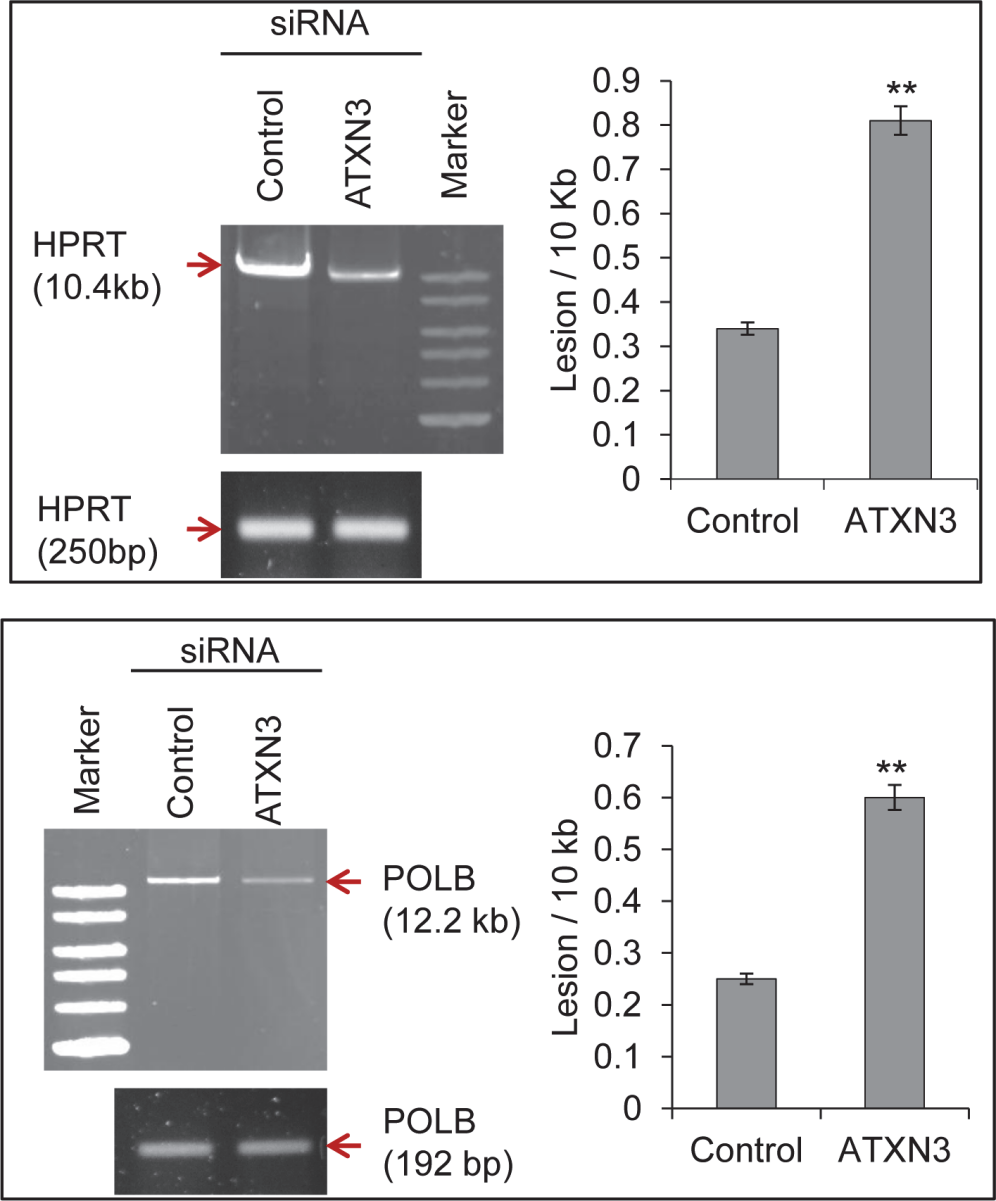
ATXN3 depletion increases DNA strand break levels in the nuclear genome. Long amplicon qPCR (LA-QPCR) was used to evaluate genomic DNA SB levels in control vs. ATXN3-depleted HEK-293 cells. Representative gel showing PCR-amplified fragments of the *HPRT* (left panel) and *POLB* (right panel) genes. Amplification of each large fragment (upper panels) was normalized to that of a small fragment of the corresponding gene (bottom panels) and the data were expressed as lesion frequency/10 Kb DNA as described previously [34]. Histograms represent the DNA damage quantitation for control vs ATXN3-depleted cells (n = 3, ** = P< 0.01). Error bars indicate standard error of means.

To examine whether the decreased repair activity of the ATXN3-depleted cells affected the accumulation of DNA strand breaks, genomic DNA was isolated from control and ATXN3-siRNA-treated cells, and the levels of SBs in the *HPRT* and *POLB* genes were compared using long amplicon quantitative PCR (LA-QPCR) as described previously [33]. DNA strand breaks were measured for both the genes using a Poisson distribution, and the results were expressed as lesion /10 kb genome [34]. Studies were conducted in both HEK-293 (Fig. 5A) as well as in neuronal SH-SY5Y cells (S8 Fig.) to examine the effect of ATXN3 depletion on DNA SB repair in general. A decreased level of the long amplicon PCR product (∼10–12 kb) would reflect a higher level of DNA SBs, and amplification of a smaller fragment for each gene should be similar for the samples, because of a lower probability of SB formation in a shorter fragment. We indeed observed a higher level of DNA lesion frequency per 10 Kb (0.81 vs 0.34 for *HPRT*, 0.6 vs 0.24 for *POLB* in HEK 293 cells; 0.45 vs 0.1 for *HPRT*, 0.38 vs 0.14 for *POLB* in SH-SY5Y cells) in the genomic DNA of ATXN3-depleted cells than in the DNA of control shRNA/siRNA-expressing cells (Fig. 5A), indicating a role of ATXN3 in DNA SB repair.

### Mutant ATXN3-expressing cells and transgenic SCA3 mouse brain tissue show decreased PNKP’s 3’-phosphatase activity

**Figure 6:**
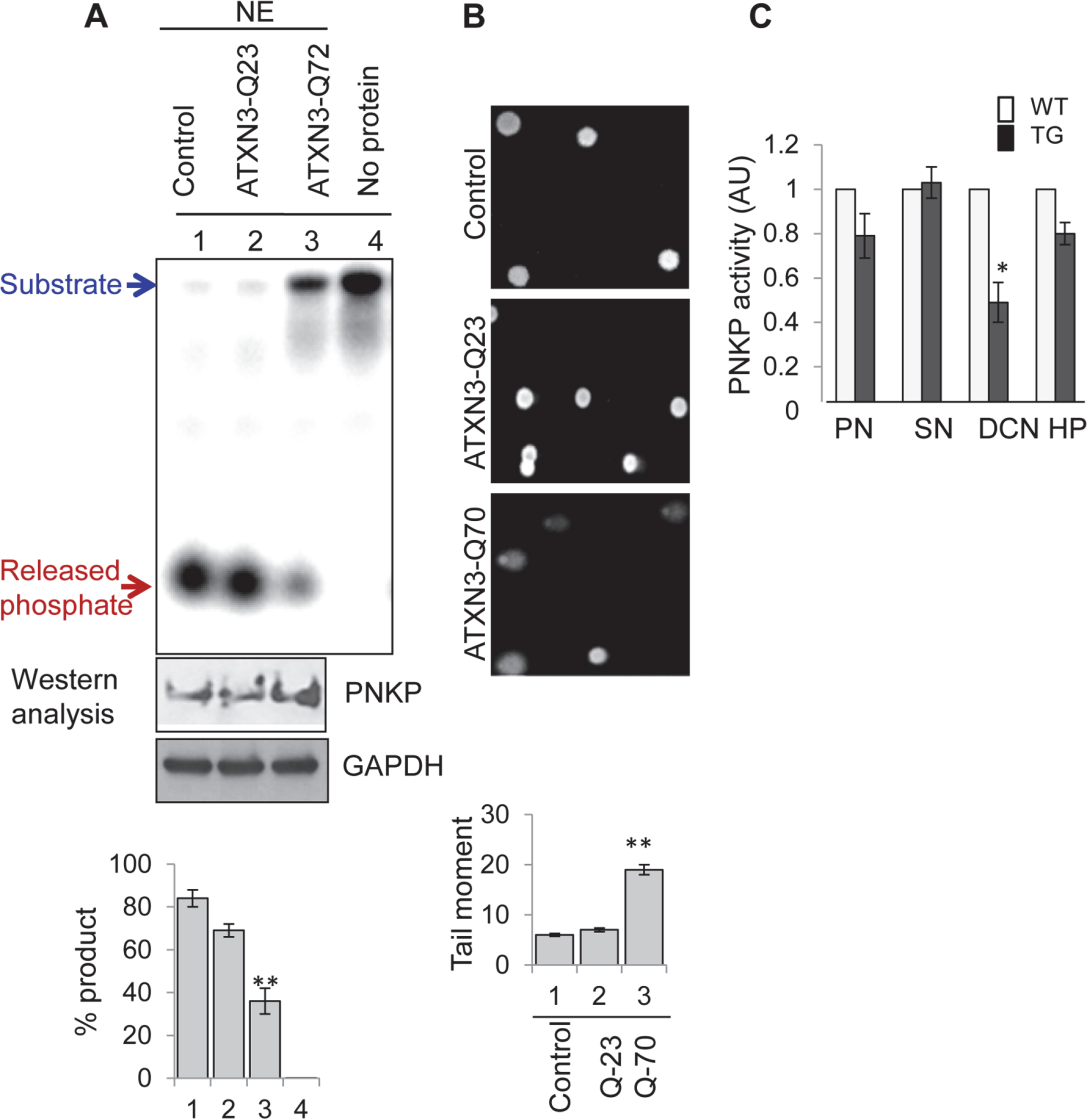
Decreased 3’-phosphatase activity in nuclear extracts from mutant ATXN3-expressing cells, and from SCA3 mouse brain regions. **(A)** Upper panel, ^32^P-labelled 3’-phosphate-containing oligo substrate (2.5 pmol) was incubated with the NE (250 ng) isolated from rat mesenchymal (CSM14.1) cells conditionally expressing vector alone (lane 1), WT (Q-23, lane 2) or mutant ATXN3 (Q-70, lane 3). Ln 4, no protein. S: substrate, P: product (released ^32^P_i_). Middle panel: Western analysis of the corresponding NE (10μg) showing PNKP expression. GAPDH (Ab from Genetex Inc.) was used as loading control. Bottom panel: quantitation of the products (P) is represented in a histogram. (n = 3, ** = P< 0.01) **(B)** Comet assays showing the accumulation of DNA SBs in mutant (Q-70) ATXN3 cells. **(C)** Comparative (WT vs. SCA3 mice) 3’-phosphatase activity in the NEs from four different brain regions. 7.5 pmol of 3’-P-containing substrate was incubated at 37°C for 10 min with WT or transgenic mouse brain NE (200 ng; PN-Pontine Nuclei, SN-Substantia Nigra, DCN-Deep Cerebellar Nuclei, and HP-Hippocampus). Quantitation of the products is shown in the histogram with the age-matched WT arbitrarily set as 1 (n = 5; ** = P< 0.01).

To further confirm the effect of pathological ATXN3 on PNKP’s activity, we measured 3’-phosphatase activity in the nuclear extracts of cells (CSM14.1, a rat cell line) conditionally expressing vector control, WT (Q23) and mutant (Q70) ATXN3[35]. Although PNKP levels were comparable in all the extracts (Fig. 6A), PNKP’s activity was significantly decreased (∼45%) in the NE of mutant ATXN3 (Q-70)-expressing compared to WT or vector control cells (Fig. 6A, ln 3 vs. lns 1, 2). We also performed comet assays to examine the level of DNA SBs, and indeed found a significant increase in tail moment with the mutant ATXN3-expressing cells (Fig. 6B), which is consistent with decreased PNKP activity and subsequent accumulation of DNA SBs. Furthermore, comet assays were conducted in PNKP-depleted and mutant ATXN3-expressing human SH-SY5Y cells and compared with the appropriately paired control cells (S9 Fig.). As expected, PNKP-depleted and mutant ATXN3-expressing cells also showed comparable amounts of DNA damage. Furthermore, we tested PNKP-mediated total single-strand break repair (SSBR) using a 3’-P-containing oligo substrate, and found that total SSBR was also significantly lower (55±4.2%) in the mutant cell NE. However, supplementation with purified PNKP rescued the repair to the level of WT extract (arbitrarily set as 100%). These data further support the idea that the mutant ATXN3 specifically blocks the activity of PNKP, but not that of DNA polymerase or ligase (S6 Fig.).

Finally, we measured PNKP’s 3’-phosphatase activity in the NEs prepared from four different regions of the brains of the transgenic mice expressing expanded human ATXN3 (CMVMJD135) vs. WT ATXN3-expressing mice [36]. Extracts were obtained from the animals at an age (25 weeks) at which they manifest loss of strength, decreased coordination of movement, loss of balance, and abnormal reflexes [37]. PNKP activity was affected in the mutant mice (n = 5), mostly in extracts obtained from the deep cerebellar nuclei, a key region known to be affected in human SCA3 neuropathology (Fig. 6C). Taken together, these results indicate that the pathological form of ATXN3 affects PNKP activity and subsequent global SB repair *in vivo*.

### Higher strand break levels in SCA3 patient samples

**Figure 7:**
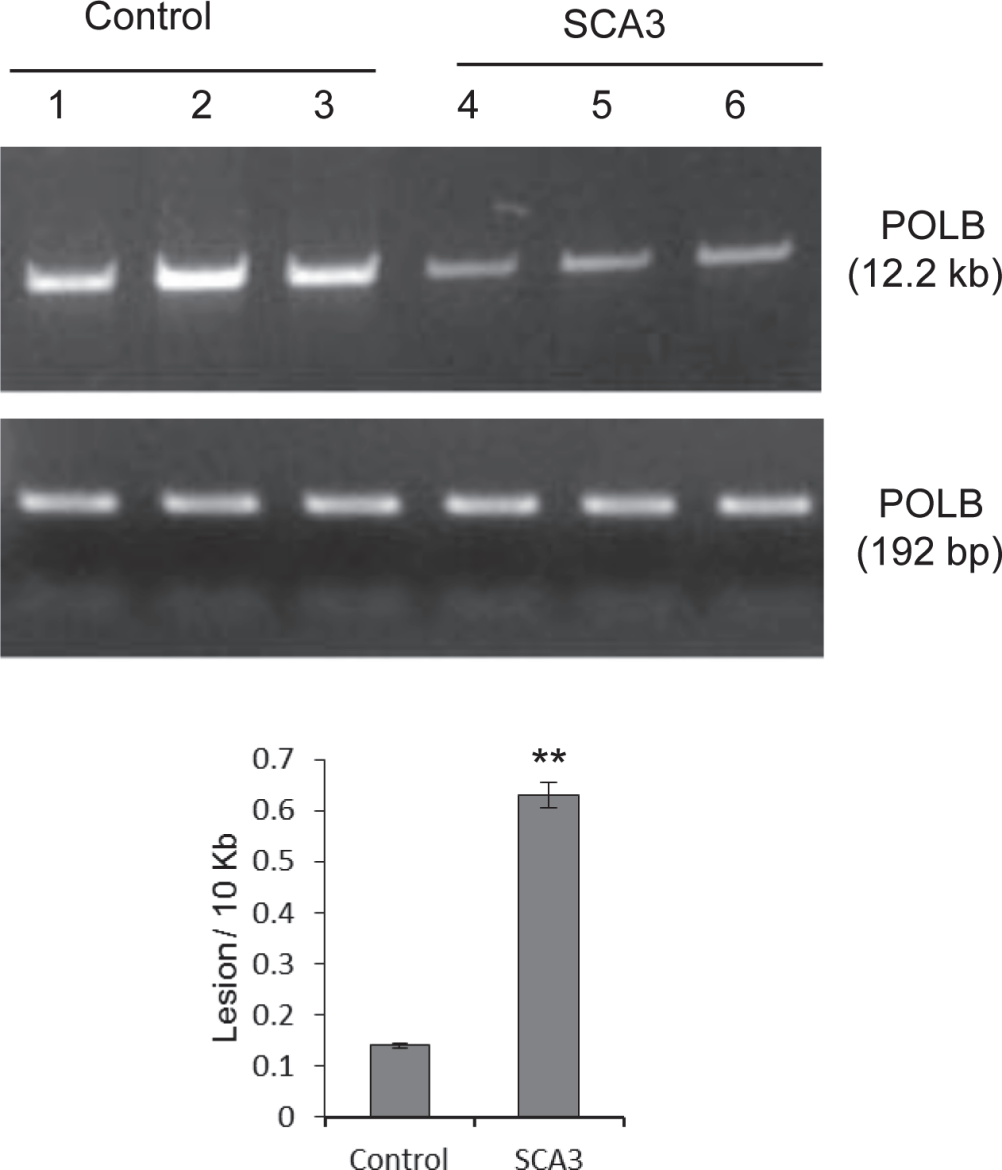
LA-QPCR shows increased genomic DNA damage levels in SCA3 patients’ brain tissue. Amplification of the large fragment (∼12 kb) of the *POLB* gene was normalized to the amplification product of a small fragment of the same gene. The results are expressed as DNA lesion frequency/10 Kb DNA by application of the Poisson distribution as described previously [34]. Histograms represent the DNA damage quantitation for age-matched control and SCA3 patient’s brain tissue (n = 3, ** = P< 0.01). Error bars indicate standard error of means.

The studies described above were helpful in understanding the basic biochemistry of the effects of WT vs. mutant ATXN3 on PNKP’s activity in cultured cells and in mouse tissues, which then prompted us to examine their relevance to human pathology. We received three SCA3 patient and age-matched control tissues from the same region of the brain (details in the accompanying manuscript by Gao et al). Although we failed to prepare intact nuclei and measure PNKP activity from post-mortem brain tissues, we were able to isolate total genomic DNA, and so analyzed the accumulation of DNA SBs in a long fragment of the *POLB* gene, using LA-QPCR as described [38]. We indeed found significantly higher levels of SBs in the genomic DNA of the SCA3 patients (0.64 vs 0.14/10 Kb) than in DNA from the control group (Fig. 7), consistent with our observations in cells and mouse tissues. Likewise, our accompanying manuscript by Gao et al. also showed the formation of 53BP1 foci, a key transducer of the DNA damage response, in SCA3 but not control brains (their Fig. 3 & S5), further supporting our hypothesis.

## Discussion

Machado-Joseph disease, or spinocerebellar ataxia type 3 (MJD/SCA3), is one of the most common dominantly inherited ataxias worldwide. Many years have passed since the cloning of ATXN3, and to date the pathogenic mechanism responsible for the disease is still not clearly understood, so no therapy is available for it. Several reports suggest that both WT and pathological forms of ATXN3 have protease, deubiquitinating (DUB), and autocatalytic activities, and are involved in the ubiquitin-proteasome pathway [39–42]. The pathological form of ATXN3 is prone to aggregation, and is thought to exert toxic effects in a dominant manner, although it has also been suggested that some loss of function of ATXN3 contributes to the disease. However, ATXN3 knockout mice have no significant SCA3-like phenotype [43], so the lack of DUB activity is not sufficient to explain the pathogenesis. Furthermore, various studies suggest that the formation of aggregates may not necessarily be required for pathological ATXN3 to exert its toxicity; aggregate formation could be a secondary phenomenon [44,45]. The relationship of inclusion bodies to cellular dysfunction in SCA3 pathogenesis thus remains controversial [44,45]. Therefore, understanding the molecular mechanism responsible for the toxic gain of function of the pathological form is important for devising ways to combat the disease. Our serendipitous and unexpected identification of ATXN3, a poly Q-containing protein with no established linkage to DNA repair, as a part of the PNKP complex prompted us to investigate ATXN3’s role in PNKP-initiated DNA end processing. Here we have clearly shown that both WT and mutant ATXN3 interact directly with PNKP. Immunohistochemical studies involving transgenic mouse tissues and human brains also showed close association of PNKP with both forms of ATXN3 (Also see Gao et al. Fig. 2 and Fig. S3, S4). However, WT ATXN3 stimulates, and by contrast mutant ATXN3 significantly blocks, PNKP’s 3’-phosphatase activity, resulting in the accumulation of DNA SBs. However, how the mutant ATXN3 blocks PNKP’s 3’-phosphatase activity, but not that of DNA polymerase β or DNA ligase IIIα, warrants further investigation. Our GST pull-down data clearly show that the kinase domain interacts with both forms, while the phosphatase domain interacts only with the WT but not the mutant ATXN3 (Fig. 2D). This aberrant interaction of the pathological form may be responsible for blocking PNKP’s 3’-phosphatase activity.

A recent study showed a role of ATXN3 in protecting cells against H_2_O_2_-induced oxidative stress [46]. The decreased 3’-phosphatase activity in the NE from ATXN3-depleted cells, which show the accumulation of SBs, and the presence of PNKP, Polβ and Lig IIIα in the ATXN3 IP further supports WT ATXN3’s role in PNKP-mediated DNA SB repair and is thus consistent with the above findings. Whether WT ATXN3 plays any role in other types of DNA end processing in association with such other enzymes as TDP1, TDP2 etc. also warrants further investigation. Furthermore, it has recently been shown that PNKP is phosphorylated by ATM (Ataxia telangiectasia mutated) and DNA-PK in response to DNA damage, which prevents degradation via ubiquitination, and the cellular level of PNKP is maintained via this process [47–49]. Whether ATXN3 plays any role in controlling PNKP’s posttranslational modification via its DUB activity, and thereby in regulating PNKP’s cellular steady-state level, is currently under investigation in our laboratory.

The human brain is one of the most metabolically active tissues and consumes large amounts of oxygen. Oxidative stress-induced DNA SBs are quite common in such tissues. DNA SBs can also arise spontaneously in the brain as a result of normal physiological neuronal activity [50]. If not repaired, DNA SBs can block transcription, which is active at all cell cycle stages and particularly important in postmitotic tissues like neurons. Recently it has been clearly documented that deficiencies in DNA end-processing activity (such as that of PNKP, TDP1 and Aprataxin) are linked to neurological diseases [1–4, 9]. Pathological ATXN3-mediated inhibition of PNKP’s activity, particularly in the context of heterozygosity (one WT and one mutant ATXN3 allele), would be a slow and progressive phenomenon, which could provide an explanation as to why the SCA3 pathology is largely related to age. We therefore hypothesize that the accumulation of persistent DNA SBs due to lower PNKP activity triggers an intrinsic signaling event leading to dysregulated neuronal gene expression that impairs neuronal function, leading to neurodegeneration and the development of ataxia. Our accompanying manuscript clearly shows that either PNKP depletion or the expression of mutant ATXN3 leads to ATM-mediated activation of two parallel proapoptotic pathways; one is p53- and the other c-Abl→PKCδ-mediated apoptotic cell death, a hallmark of neuropathogenesis (Gao et al. Figs. 4–7, Figures S15–S17). These data thus provided further support for our hypothesis. It is noteworthy that persistent accumulation of DNA SBs and elevated p53-dependent apoptosis have also been found to be an important pathogenic feature in Fragile X syndrome (one of the most common form of inherited mental retardation), which is caused by the transcriptional silencing of fragile X mental retardation protein (FMRP;[51]). FMRP is primarily localized in the cytoplasm; however, a small amount is present in the nucleus, which raised the possibility that the protein might have a role there as well. Recent studies involving both *Drosophila* and mammalian systems showed a novel and unanticipated role of nuclear FMRP protein, which takes part in the DNA Damage Response (DDR) pathway via γH2AX phosphorylation in a chromatin binding-dependent manner [52,53]. In a separate report it was also shown, that a *Drosophila* FMRP mutant (*dfmr1*) is hypersensitive to genotoxic stress [51]. Collectively, all these data reflect a degree of mechanistic similarity between ATXN3 and FMRP in regulating DDR, and their subsequent role in neuronal survival via maintaining genome stability. Repeat expansions and instabilities are causal factors for numerous inherited neurological disorders, including SCAs [54,55]. A recent report showed elevated expression levels of DNA repair proteins in the cerebellum compared to the striatum, and consequent higher repeat instability in the striatum as well. These data suggest that higher level/activity of DNA repair proteins act as a safeguard against repeat instability, which in turn leads to reduced somatic genome instability in the specific region of brain dictating the related disease pathologies [56].

In conclusion, MJD/SCA3 is a complex disease; modulation of various cellular processes, such as protein aggregation and loss of DUB activity may all play a role in the disease process. Our studies show that the accumulation of DNA SBs due to decreased PNKP activity is likely to be the major contributor to SCA3 pathogenesis. Therefore, upregulating the expression of PNKP might be a promising therapeutic strategy for combating SCA3 pathogenesis.

## Materials and Methods

### Cell culture, siRNA transfection and generation of stable cell line

Human gastric epithelial AGS (purchased from ATCC) and human embryonic kidney (HEK-293) cells were cultured and maintained in DMEM/F12 containing 10% FBS at 37°C. Mesencephalic dopaminergic rat cell lines (CSM14.1, a gift from Bernd Evert, University of Bonn) conditionally expressing vector alone or WT human ATXN3 (Q23) or pathological ATXN3 (Q70) were cultured and maintained at 33°C in DMEM with 10% FBS containing 0.1 mg/ml G418, 0.1 mg/ml hygromycin, 4.0 ug/ml puromycin and 1.0 μg/ml tetracycline as described previously [35].

The human neuroblastoma cell line SH-SY5Y (ATCC number CRL-2266) was cultured at 37°C in a 1:1 mixture of DMEM/high glucose nutrient (Invitrogen) supplemented with 10% (v/v) fetal bovine serum (FBS) (Biochrom), 2 mM glutaMAX (Invitrogen), 100 U/mL penicillin and 100 μg/mL streptomycin. To generate ATXN3-depleted cells, SH-SY5Y cells were transfected with an shRNA sequence targeting *ATXN3* or a scrambled shRNA sequence, as described elsewhere and the stably transduced cells were selected 48 h post-transfection with 500 ng/ml puromycin. Stably infected cell lines were cultured and maintained as described earlier in presence of 25 ng/mL puromycin (Sigma Aldrich). The medium was changed every two days. Differentiation was induced by exposure to 0.1 μM all-trans-retinoic acid (RA, Sigma Aldrich) in opti-MEM (Invitrogen) supplemented with 0.5% FBS for 7 days; the medium was replaced every two days.

PNKP and ATXN3 depletion was carried out in HEK-293 cells using siRNAs (80 nM) purchased from Sigma (SASI_Hs01_00067475) and Dharmacon (On-target siRNA, J-012013-05), respectively. The control siRNA was purchased from Sigma (Mission universal control, SIC001). The cells were harvested 60 h post-transfection and nuclear extracts were prepared as described [57].

### Analysis of PNKP-associated proteins

A large-scale immunoprecipitation from AGS (gastric epithelial) cell nuclear extracts (100 mg, benzonase treated to remove DNA and RNA to avoid DNA-mediated co-immunoprecipitation) used mouse IgG (control) and anti-PNKP antibody (mouse monoclonal Ab, Cytostore)- conjugated agarose beads as described earlier [58]. The immunoprecipitates (IPs) were washed extensively with cold TBS (50 mM Tris-HCl, pH 7.5; 200 mM NaCl) containing 1 mM EDTA, 1% Triton X-100 and 10% glycerol. The complexes were then eluted from the beads stepwise with 25 mM Tris-HCl, pH 7.5 containing 300, 400 and 500 mM NaCl. The eluates were subjected to 2-dimensional gel electrophoresis (2-DE) separation and the protein spots (Sypro Ruby, Molecular Probes) that were specifically present in the PNKP IP and not in the IgG IP were subjected to mass spectroscopic identification in the University of Texas Medical Branch Biomolecular Resource Facility.

Co-IP analysis was performed from HEK-293 and SH-SY5Y cells according to established protocol by Aygun et al, with modifications as applicable [57].

*In situ* Proximity Ligation Assay (PLA) between PNKP (anti-mouse Ab, a gift from Michael Weinfeld) and ATXN3 (anti-rabbit Ab, Proteintech) was carried out according to the protocol as described [58] using a Duolink PLA kit (QLink Bioscience Cat# LNK 92101 K101, Uppsala, Sweden).

### 3’-phosphatase activity of PNKP

The 3’-phosphatase activity of PNKP in the nuclear extract or with purified recombinant PNKP was assayed as we described previously [58,59].

### DNA damage quantitation by long amplicon quantitative PCR (LA-QPCR)

Genomic DNA from HEK-293 and SH-SY5Y cells was extracted using the Qiagen Genomic-tip 20/G kit per the manufacturer’s directions. This kit is particularly useful, as it minimizes DNA oxidation during the isolation step and has been previously used for LA-QPCR assays [33,38]. For isolation of genomic DNA from postmortem brain tissues, we followed the protocol of Kovtun et al. as described [60]. To decrease aerial oxidation during genomic DNA preparation, TEMPO (2,2,6,6-tetramethylpiperidine-*N*-oxyl) was added to all solutions at a concentration of 100μM immediately before use [60]. The DNA was quantitated by Pico Green (Molecular Probes) in a 96-well plate. Gene-specific LA-QPCR assays for measuring DNA SBs were performed as described earlier [33] using LongAmp Taq DNA Polymerase (New England BioLabs). A 10.4 kb region of the *HPRT* gene or 12.2 kb of the *POLB* gene was amplified from human genomic DNA using the primers described previously [38]. To ensure the linearity of PCR amplification with respect to the number of cycles and DNA concentration, preliminary assays were carried out. Since amplification of a small region would be independent of DNA damage, a small DNA fragment from the same gene was also amplified for normalization of amplification of the large fragment [33]. The amplified products were then visualized on gels and quantitated with ImageJ software system. The extent of damage was calculated in terms of lesion/10 kb genome following Poisson’s distribution according to methods as described [34].

### Protein purification

We received the bacterial expression vectors for ATXN3-Q29 (WT) and ATXN3-Q72 (mutant) as a kind gift from Randall Pittman (Univ. of Pennsylvania) and purified both recombinant proteins as described[19,20]. WT PNKP and its domains were purified as described previously [58]. Purified fractions were dialyzed in PBS containing 50% glycerol and 1 mM DTT and stored at −20°C.

### GST pull-down

GST pulldown assays were performed as described previously [61]. Briefly, GST-tagged full-length PNKP or its three individual domains (20 pmol) were bound to glutathione-Sepharose beads (20 μL), washed thoroughly with buffer A (25 mM Tris-Cl pH 7.5, 0.1% Triton X-100, 0.1 mM EDTA and 10% glycerol) containing 150 mM NaCl, and then incubated with WT or mutant ATXN3 (20 pmol) with constant rocking for 4 h. at 4°C in 0.5 ml of 150 mM salt containing buffer A. After extensive washing with 200 mM NaCl containing buffer A, 20% of the bound proteins were separated by SDS-PAGE for immunoblotting analysis using an anti-ATXN3 Ab (Abcam).

### SCA3 mice

CMVMJD135 mice, expressing human ataxin-3 carrying 135 glutamines, were used in this study [37]. These mice display a progressive motor phenotype starting at an age of 6 weeks, with extensive phenotypic overlap with the human disease; they also develop ATXN3-positive neuronal inclusions in different regions of the brain and spinal cord, as well as a cell number and/or volume decrease in key regions for the disease, such as the pontine nuclei and the dentate nuclei of the cerebellum. Transgenic mice and control non-transgenic littermate mice (n = 5 per genotype) with a mean age of 25 weeks were sacrificed by decapitation, and brain slices were obtained for the macrodissection of pontine nuclei, substantia nigra, deep cerebellar nuclei and hippocampi using a stereomicroscope (Model SZX7, Olympus America Inc., Center Valley, PA, USA). Nuclear extracts from these different brain regions were obtained as previously described [62].

## Supporting Information

S1 Fig Identification of ATXN3 in the PNKP IP by MS analysis. The upper panel shows the peptide sequence coverage after MS/MS analysis, with eight peptide sequences (underlined) exactly matching the human ATXN3 protein sequence. The lower panel shows a summary of the original MS data in tabular form.
(TIF)

S2 Fig PNKP and ATXN3 associate in SH-SY5Y human neuroblastoma cells. (**A)** Characterization of the PNKP immunocomplex by Western blot analysis. Nuclear extracts (1 mg) from SH-SY5Y cells were IP’d with anti-PNKP antibody (Ab, BioBharati Life Science Pvt. Ltd, Kolkata, India) and tested for the presence of ATXN3 and PNKP-associated proteins with Abs to the proteins shown on the right. **(B)** Endogenous ATXN3 from the nuclear extract of SH-SY5Y cells was IP’d using IgG or anti-ATXN3 Ab (Proteintech) and tested for the presence of PNKP, Polβ and Lig IIIα.
(TIF)

S3 Fig siRNA-mediated depletion of PNKP. **(A)** Coomassie-stained gel showing equal loading of NE (25 μg) from control and PNKP siRNA depleted HEK-293 cells. **(B)** A second gel was run in parallel for Western analysis to confirm specific depletion of PNKP (lane 7, Left panel). GAPDH is used as a loading control (right panel). Purified PNKP (25 ng) is used as a marker.
(TIF)

S4 Fig siRNA-mediated depletion of ATXN3. **(A)** Coomassie-stained gel showing equal loading of NE (25 μg) from control and ATXN3 siRNA depleted HEK-293 cells. **(B)** Western analysis ( 2^nd^ gel ) to confirm specific depletion of ATXN3 (lane 6, Left panel). GAPDH is used as a loading control (right panel). Purified ATXN3 (Q-29, 25 ng) is used as a marker.
(TIF)

S5 Fig Far-western analysis shows interaction of PNKP with both WT and mutant ATXN3. Top panel, far-Western [53] showing interaction of PNKP with wild-type (ln 1) and mutant ATXN3 (ln 2), and BSA (negative control; ln 3). Bottom panel: Coomassie staining of a 2^nd^ gel run in parallel.
(TIF)

S6 Fig ATXN3 (WT or mutant) has no effect on DNA polymerase and ligase activities. **(A)** Pol β (50 fmol) activity was measured in the presence of increasing amounts (50 and 100 fmol) of Q72 (lns 2, 3) or Q29 (lns 4, 5) ATXN3, using an oligo substrate (0.5 pmol) generated by annealing a 25-nt oligo with a 51-nt complementary strand. The assay is based on a single-turnover reaction, monitored by examining the incorporation of [α-^32^P]-dTMP at the 3’ end of a 25-mer primer as shown at the top of the figure. **(B)** DNA ligase IIIα activity was measured in the presence of increasing amounts (50 and 100 fmol) of Q29 (lns 3, 4) or Q72 (lns 5, 6) ATXN3, using an oligo substrate (0.5 pmol) generated by annealing two oligos 25 nt (^32^P-labelled at the 5’-end) and 26 nt long (phosphorylated at the 5’-end) with a 51-nt complementary strand, as shown at the top of the figure.
(TIF)

S7 Fig Effect of WT (Q-29) and mutant ATXN3 (Q-72) on the 3’phosphatase activity in the nuclear extract. ^32^P-labelled 3’-phosphate-containing oligo substrate (5 pmol) was incubated at 37°C for 10 min in buffer A (25 mM Tris-HCl, pH 7.5, 100 mM NaCl, 5 mM MgCl_2_, 1 mM DTT, 10% glycerol and 0.1 μg/μl acetylated BSA) with NE (250 ng) prepared from control (ln 1) and PNKP siRNA treated HEK 293 cells (ln 2). Lns 3 and 4, purified (100 fmol) wild type (Q-29) and mutant (Q-72) ATXN3 respectively was added back to the PNKP depleted NE. Ln 5, purified PNKP (25 fmol) was used as a positive control for released phosphate, as a marker. Ln 6, γ^32^P-ATP, to show that it’s migration is slower than free phosphate. Ln 7, no protein control with higher substrate amount (15 pmol) to show the absence of non-specific radioactive bands in the substrate preparation.
(TIF)

S8 Fig ATXN3 depletion increases DNA strand break levels in the nuclear genome. Long amplicon qPCR (LA-QPCR) was used to evaluate genomic DNA SB levels in control vs. ATXN3-depleted SH-SY5Y cells. Representative gel showing PCR-amplified fragments of the *HPRT* (left panel) and *POLB* (right panel) genes. Amplification of each large fragment (upper panels) was normalized to that of a small fragment of the corresponding gene (bottom panels). Lesion frequency/10 Kb DNA was measured using Poisson distributions as described previously [34]. Histograms represent the DNA damage quantitation for control vs ATXN3 depleted cells (n = 3, ** = P< 0.01). Error bars indicate standard error of means.
(TIF)

S9 Fig Targeted depletion of PNKP in SH-SY5Y cells induces DNA damage. (Upper panel), Comet assay of SH-SY5Y cells transfected with control-siRNA vs. cells transfected with PNKP-siRNA (200 pmoles); the comet tails indicating DNA damage are shown with arrows. Bar diagram shows relative DNA damage/fragmentation in cells treated with control-siRNA vs. cells treated with PNKP-siRNA, n = 100, data represents mean ± SD, *** = p<0.001. Expression of mutant ATXN3 in SH-SY5Y cells induces DNA damage (Lower panel). Single-cell gel electrophoresis (comet assay) of SH-SY5Y cells expressing mutant ATXN3 and wild-type ATXN3 (comet tails indicating DNA damage are shown by arrows). Bar diagram shows relative DNA damage/fragmentation (expressed as comet tail moment) in SH-SY5Y cells expressing wild-type ATXN3 vs. mutant ATXN3, n = 100, data represents mean ± SD; *** = p<0.001.
(TIF)
