# Correction: The Role of the Mammalian DNA End-processing Enzyme Polynucleotide Kinase 3’-Phosphatase in Spinocerebellar Ataxia Type 3 Pathogenesis

**DOI:** 10.1371/journal.pgen.1011124

**Published:** 2024-01-18

**Authors:** Arpita Chatterjee, Saikat Saha, Anirban Chakraborty, Anabela Silva-Fernandes, Santi M. Mandal, Andreia Neves-Carvalho, Yongping Liu, Raj K. Pandita, Muralidhar L. Hegde, Pavana M. Hegde, Istvan Boldogh, Tetsuo Ashizawa, Arnulf H. Koeppen, Tej K. Pandita, Patricia Maciel, Partha S. Sarkar, Tapas K. Hazra

After this article [1] was published, concerns were raised that the PNKP panel in Fig 2A appears similar to the PNKP panel in Fig 2B when the aspect ratio is adjusted.

In response, the corresponding author stated that the PNKP panels in Fig 2A and 2B show apparent resemblance, but are not duplicates, and they were derived from two separate gels representing different experimental conditions and developed on the same X-ray film. The PLOS Editors consider the concern resolved following assessment of the data files (S1 File).

In preparing the panel for purified full-length and individual PNKP domains in Fig 2D, a lane with purified full length PNKP was removed between lanes two and three (original Coomassie stained SDS-PAGE image is in S2 File). An updated version of Fig 2 is presented here to clearly indicate the cut position with a vertical black line.

Underlying data for results reported in this article are in S1–S8 Files. The image data underlying the PNKP panels in Fig 2A–2B, Fig 2D, the autoradiographic images in Figs 3A-B and 4B, the top panel in Fig 6A, and the autoradiographic image in S6B Fig have been reviewed by PLOS.

The corresponding author stated that the individual-level data underlying S6 and S9 Figs are no longer available.

**Fig 2:**
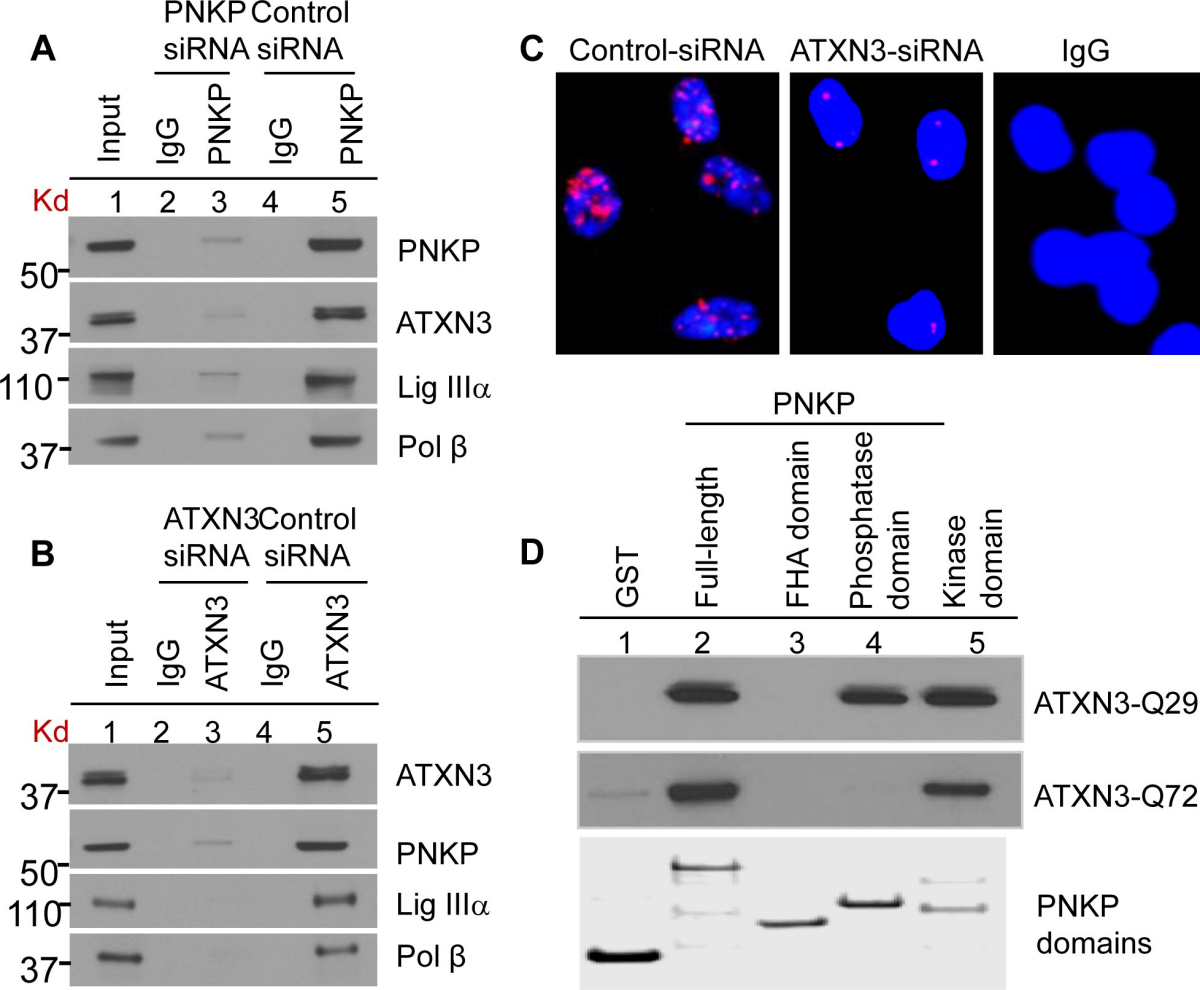
Characterization of the (A) PNKP and (B) ATXN3 immunocomplexes by Western blot analysis. HEK-293 cells were transfected with either PNKP siRNA or ATXN3 siRNA or control siRNA and the nuclear extracts (1 mg) prepared from those cells were IP’d with anti-PNKP Ab (BioBharati Life Science Pvt. Ltd, Kolkata, India; **2A**) or anti-ATXN3 Ab (Proteintech, **2B**) or with IgG as a control, and tested for the presence of PNKP- and ATXN3- associated proteins with Abs to the proteins shown on the right. **(C)** Detection of PNKP’s interaction with ATXN3 in SH-SY5Y cells by proximity ligation assays using a Duolink kit (Olink Bioscience, Uppsala, Sweden). Nuclei were counterstained with DAPI (blue). ATXN3-depleted (by siRNA) cells were used as a control to show the specificity of the interaction (middle panel). Right panel, Non-specific Ab (IgG) control **(D)** GST-PNKP pull-down of ATXN3 (WT and mutant) using purified GST-tagged full-length or three domains (FHA-, Phosphatase- and Kinase-domain) of PNKP, probed with anti-ATXN3 antibody. Bottom panel, Coomassie-stained gel of the corresponding PNKP domains, a second gel run in parallel. A replicate lane, showing full length purified PNKP (between lanes 2 and 3), was cut to avoid repetition and the remaining gel sections were joined together (indicated by a black solid line). The spliced sections originated from the same gel.

## Supporting information

S1 File Underlying blots for the panels in Fig 2A and 2B. (PPTX)

S2 File Underlying Coomassie stained SDS-PAGE image for Fig 2D. (PPTX)

S3 File Underlying autoradiograms for the panels in Figs 3A and B. (PPTX)

S4 File Underlying autoradiogram for the panel in Fig 4B. (PPTX)

S5 File Underlying autoradiogram and blots for the panels in Fig 6A. (PPTX)

S6 File Underlying autoradiograms for the panels in Figs S6A and B. (PPTX)

S7 File Individual-level underlying quantitative data for Figs 3A-B, 4B, and 6A-C, and average values for Fig S6. (XLSX)

S8 File Individual-level underlying quantitative data for Fig 6B. (XLSX)
