# Supplementary figures and images for: The Role of the Mammalian DNA End-processing Enzyme Polynucleotide Kinase 3’-Phosphatase in Spinocerebellar Ataxia Type 3 Pathogenesis
Source: PLoS Genet. 2015 Jan 29;11(1):e1004749. doi: 10.1371/journal.pgen.1004749 (PMC4310589; doi:10.1371/journal.pgen.1004749)

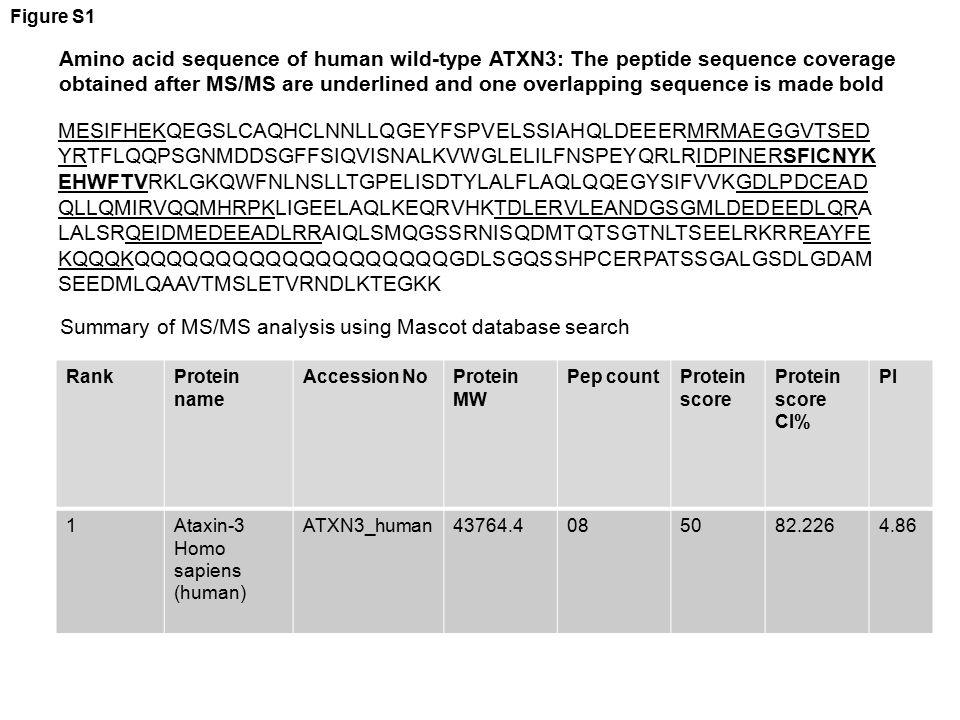

Supplement: S1 Fig — The upper panel shows the peptide sequence coverage after MS/MS analysis, with eight peptide sequences (underlined) exactly matching the human ATXN3 protein sequence. The lower panel shows a summary of the original MS data in tabular form. (TIF) [file pgen.1004749.s001.tif]

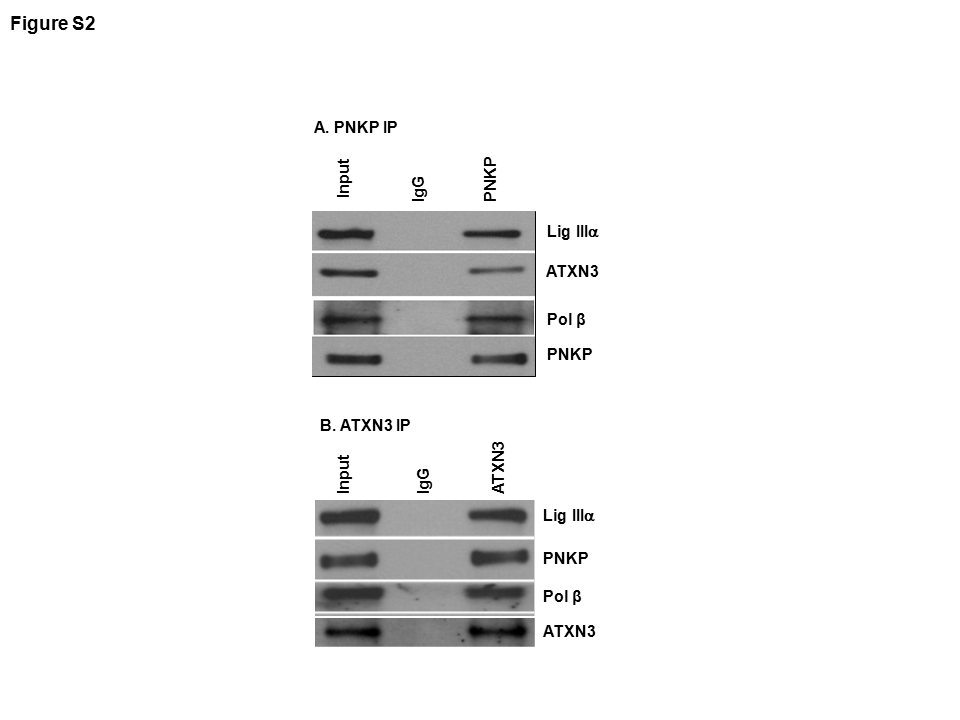

Supplement: S2 Fig — (A) Characterization of the PNKP immunocomplex by Western blot analysis. Nuclear extracts (1 mg) from SH-SY5Y cells were IP’d with anti-PNKP antibody (Ab, BioBharati Life Science Pvt. Ltd, Kolkata, India) and tested for the presence of ATXN3 and PNKP-associated proteins with Abs to the proteins shown on the right. (B) Endogenous ATXN3 from the nuclear extract of SH-SY5Y cells was IP’d using IgG or anti-ATXN3 Ab (Proteintech) and tested for the presence of PNKP, Polβ and Lig IIIα. (TIF) [file pgen.1004749.s002.tif]

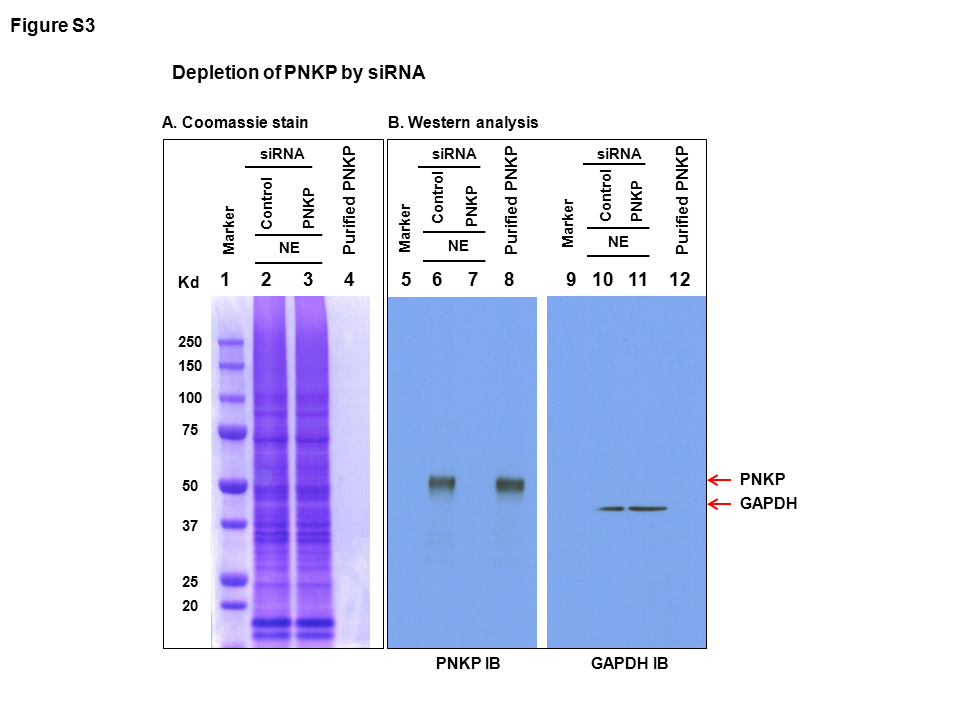

Supplement: S3 Fig — (A) Coomassie-stained gel showing equal loading of NE (25 μg) from control and PNKP siRNA depleted HEK-293 cells. (B) A second gel was run in parallel for Western analysis to confirm specific depletion of PNKP (lane 7, Left panel). GAPDH is used as a loading control (right panel). Purified PNKP (25 ng) is used as a marker. (TIF) [file pgen.1004749.s003.tif]

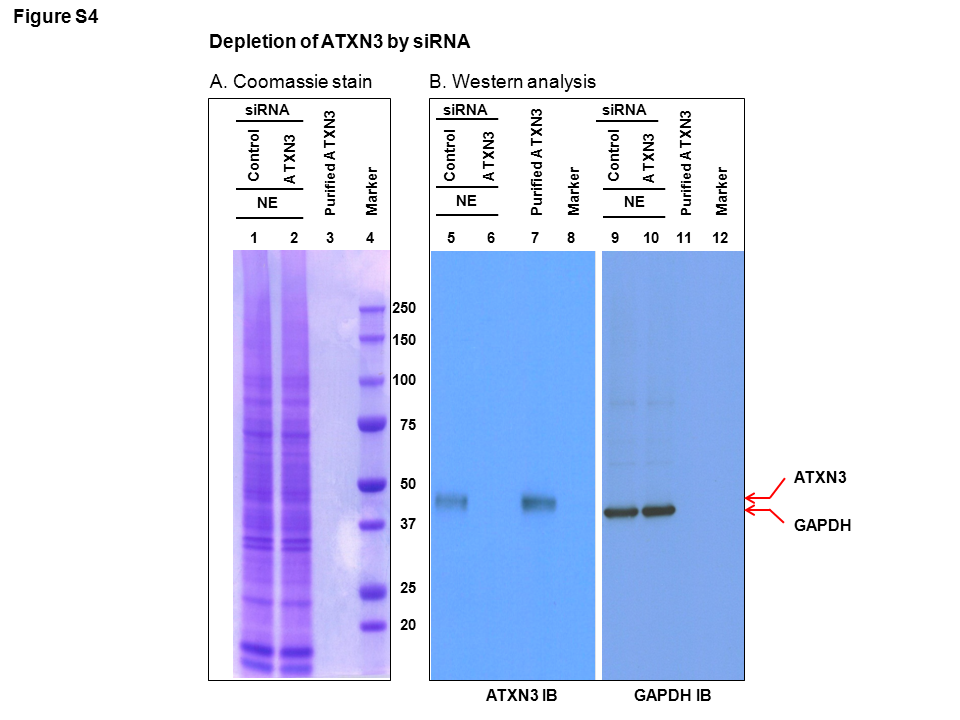

Supplement: S4 Fig — (A) Coomassie-stained gel showing equal loading of NE (25 μg) from control and ATXN3 siRNA depleted HEK-293 cells. (B) Western analysis ( 2nd gel ) to confirm specific depletion of ATXN3 (lane 6, Left panel). GAPDH is used as a loading control (right panel). Purified ATXN3 (Q-29, 25 ng) is used as a marker. (TIF) [file pgen.1004749.s004.tif]

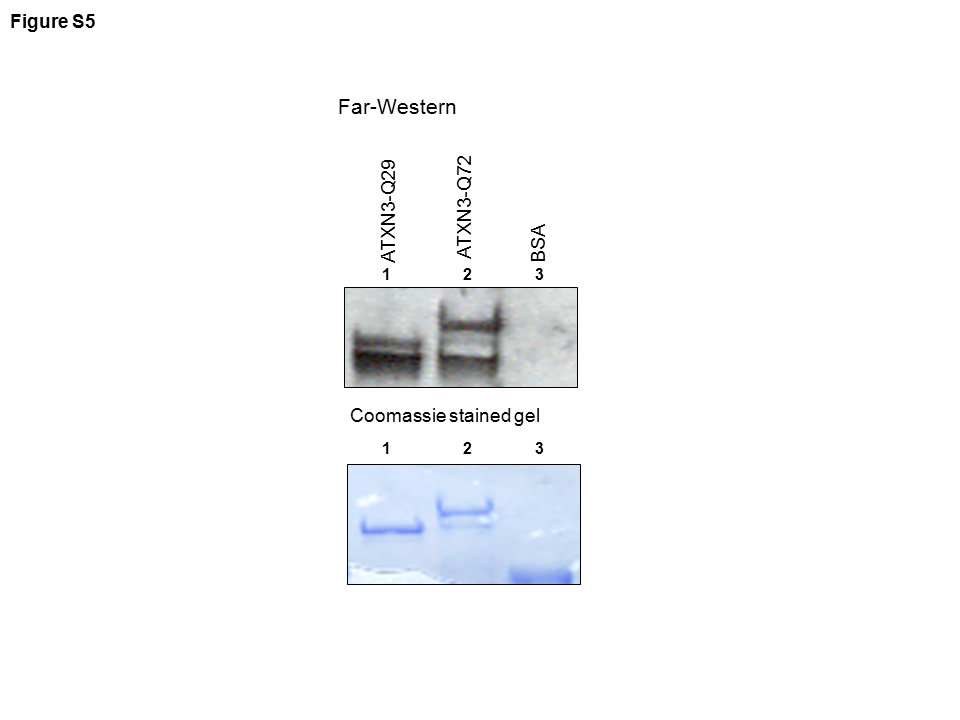

Supplement: S5 Fig — Top panel, far-Western [53] showing interaction of PNKP with wild-type (ln 1) and mutant ATXN3 (ln 2), and BSA (negative control; ln 3). Bottom panel: Coomassie staining of a 2nd gel run in parallel. (TIF) [file pgen.1004749.s005.tif]

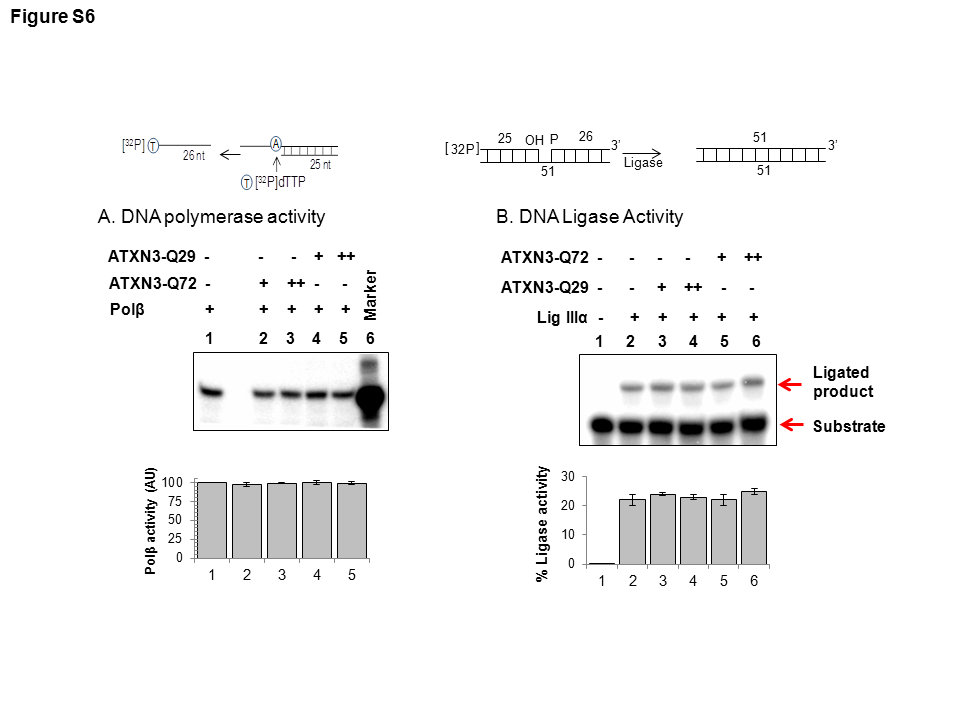

Supplement: S6 Fig — (A) Pol β (50 fmol) activity was measured in the presence of increasing amounts (50 and 100 fmol) of Q72 (lns 2, 3) or Q29 (lns 4, 5) ATXN3, using an oligo substrate (0.5 pmol) generated by annealing a 25-nt oligo with a 51-nt complementary strand. The assay is based on a single-turnover reaction, monitored by examining the incorporation of [α-32P]-dTMP at the 3’ end of a 25-mer primer as shown at the top of the figure. (B) DNA ligase IIIα activity was measured in the presence of increasing amounts (50 and 100 fmol) of Q29 (lns 3, 4) or Q72 (lns 5, 6) ATXN3, using an oligo substrate (0.5 pmol) generated by annealing two oligos 25 nt (32P-labelled at the 5’-end) and 26 nt long (phosphorylated at the 5’-end) with a 51-nt complementary strand, as shown at the top of the figure. (TIF) [file pgen.1004749.s006.tif]

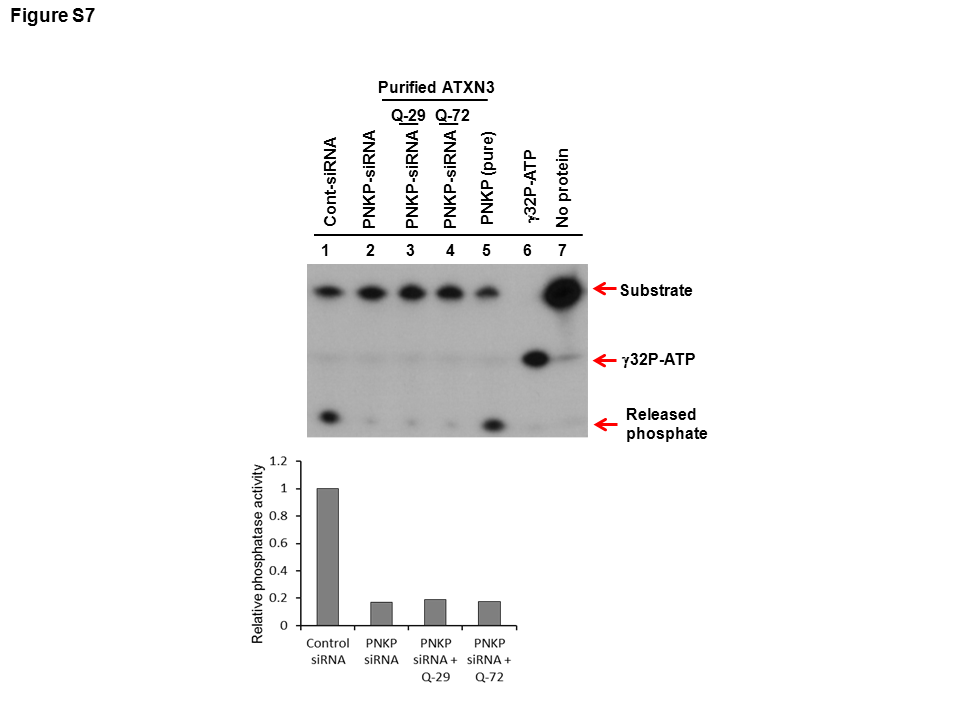

Supplement: S7 Fig — 32P-labelled 3’-phosphate-containing oligo substrate (5 pmol) was incubated at 37°C for 10 min in buffer A (25 mM Tris-HCl, pH 7.5, 100 mM NaCl, 5 mM MgCl2, 1 mM DTT, 10% glycerol and 0.1 μg/μl acetylated BSA) with NE (250 ng) prepared from control (ln 1) and PNKP siRNA treated HEK 293 cells (ln 2). Lns 3 and 4, purified (100 fmol) wild type (Q-29) and mutant (Q-72) ATXN3 respectively was added back to the PNKP depleted NE. Ln 5, purified PNKP (25 fmol) was used as a positive control for released phosphate, as a marker. Ln 6, γ32P-ATP, to show that it’s migration is slower than free phosphate. Ln 7, no protein control with higher substrate amount (15 pmol) to show the absence of non-specific radioactive bands in the substrate preparation. (TIF) [file pgen.1004749.s007.tif]

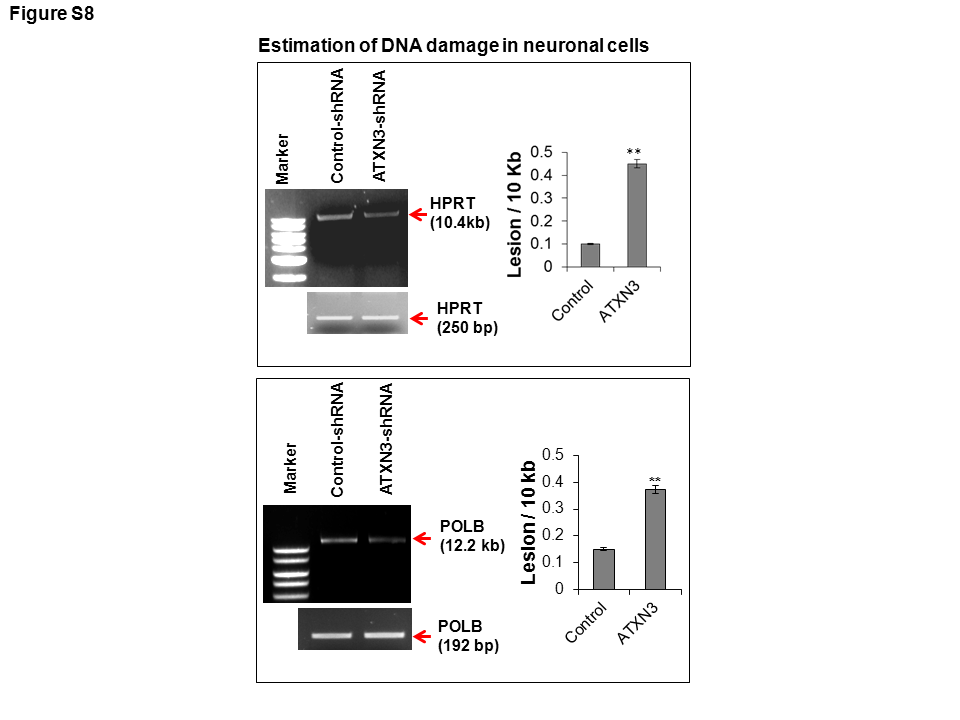

Supplement: S8 Fig — Long amplicon qPCR (LA-QPCR) was used to evaluate genomic DNA SB levels in control vs. ATXN3-depleted SH-SY5Y cells. Representative gel showing PCR-amplified fragments of the HPRT (left panel) and POLB (right panel) genes. Amplification of each large fragment (upper panels) was normalized to that of a small fragment of the corresponding gene (bottom panels). Lesion frequency/10 Kb DNA was measured using Poisson distributions as described previously [34]. Histograms represent the DNA damage quantitation for control vs ATXN3 depleted cells (n = 3, ** = P< 0.01). Error bars indicate standard error of means. (TIF) [file pgen.1004749.s008.tif]

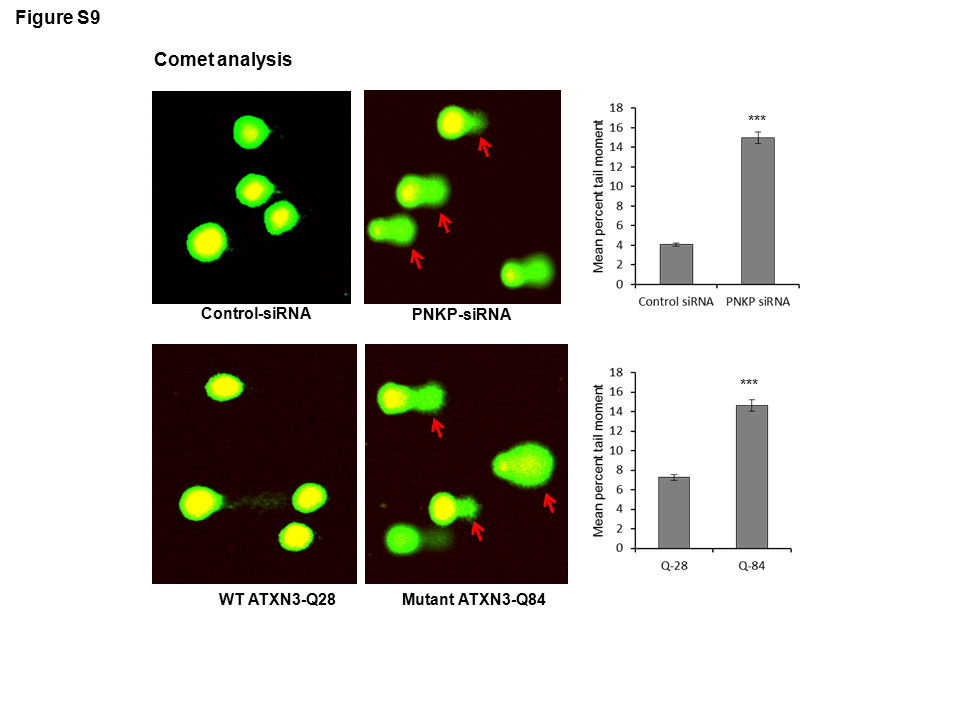

Supplement: S9 Fig — (Upper panel), Comet assay of SH-SY5Y cells transfected with control-siRNA vs. cells transfected with PNKP-siRNA (200 pmoles); the comet tails indicating DNA damage are shown with arrows. Bar diagram shows relative DNA damage/fragmentation in cells treated with control-siRNA vs. cells treated with PNKP-siRNA, n = 100, data represents mean ± SD, *** = p<0.001. Expression of mutant ATXN3 in SH-SY5Y cells induces DNA damage (Lower panel). Single-cell gel electrophoresis (comet assay) of SH-SY5Y cells expressing mutant ATXN3 and wild-type ATXN3 (comet tails indicating DNA damage are shown by arrows). Bar diagram shows relative DNA damage/fragmentation (expressed as comet tail moment) in SH-SY5Y cells expressing wild-type ATXN3 vs. mutant ATXN3, n = 100, data represents mean ± SD; *** = p<0.001. (TIF) [file pgen.1004749.s009.tif]
