# Supplementary material for: Correction: The Role of the Mammalian DNA End-processing Enzyme Polynucleotide Kinase 3’-Phosphatase in Spinocerebellar Ataxia Type 3 Pathogenesis
Source: PLoS Genet. 2024 Jan 18;20(1):e1011124. doi: 10.1371/journal.pgen.1011124 (PMC10795974; doi:10.1371/journal.pgen.1011124)
Supplement: S1 File — (PPTX) [file pgen.1011124.s001.pptx]

## Slide 1
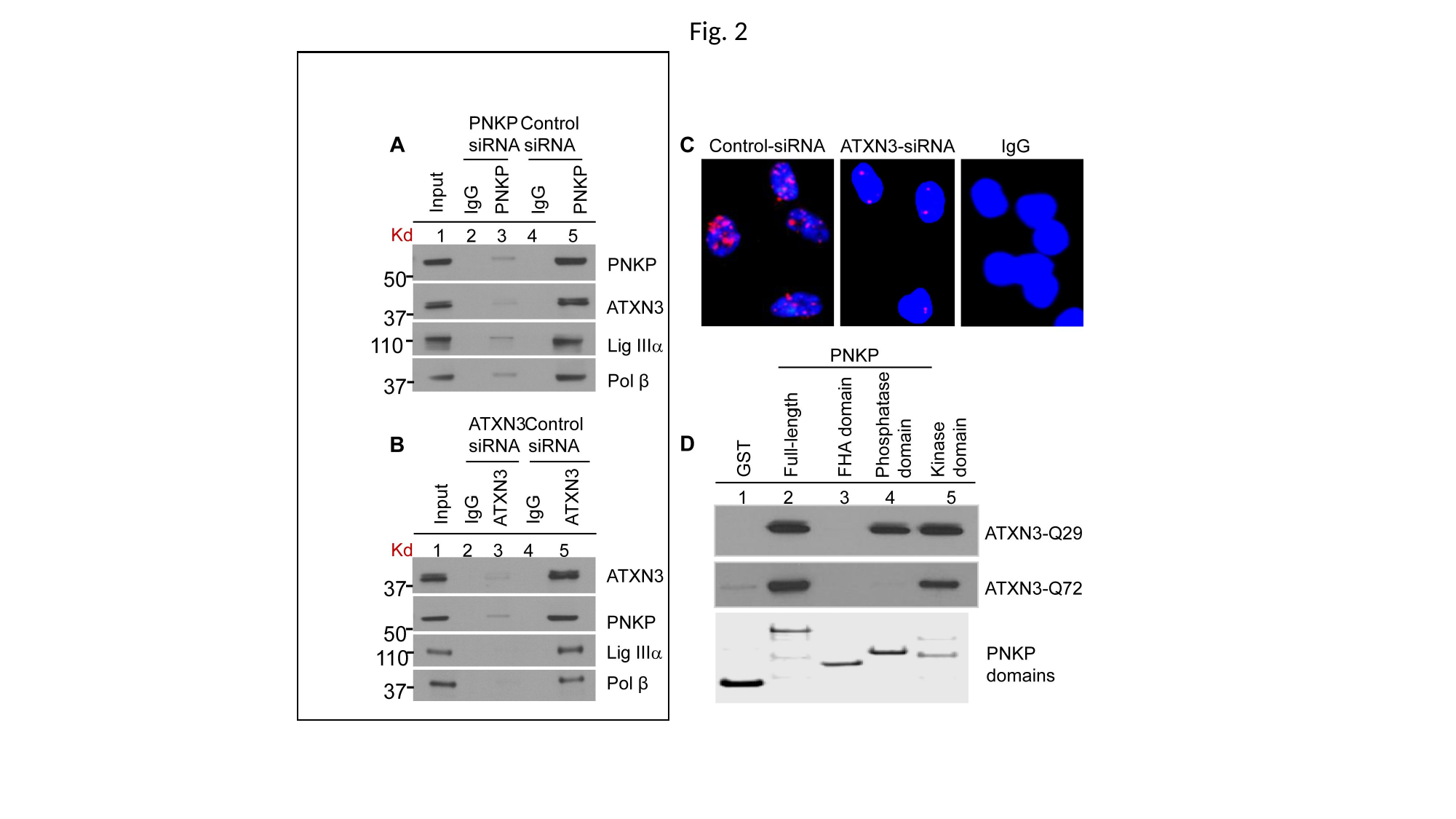

Fig. 2

## Slide 2
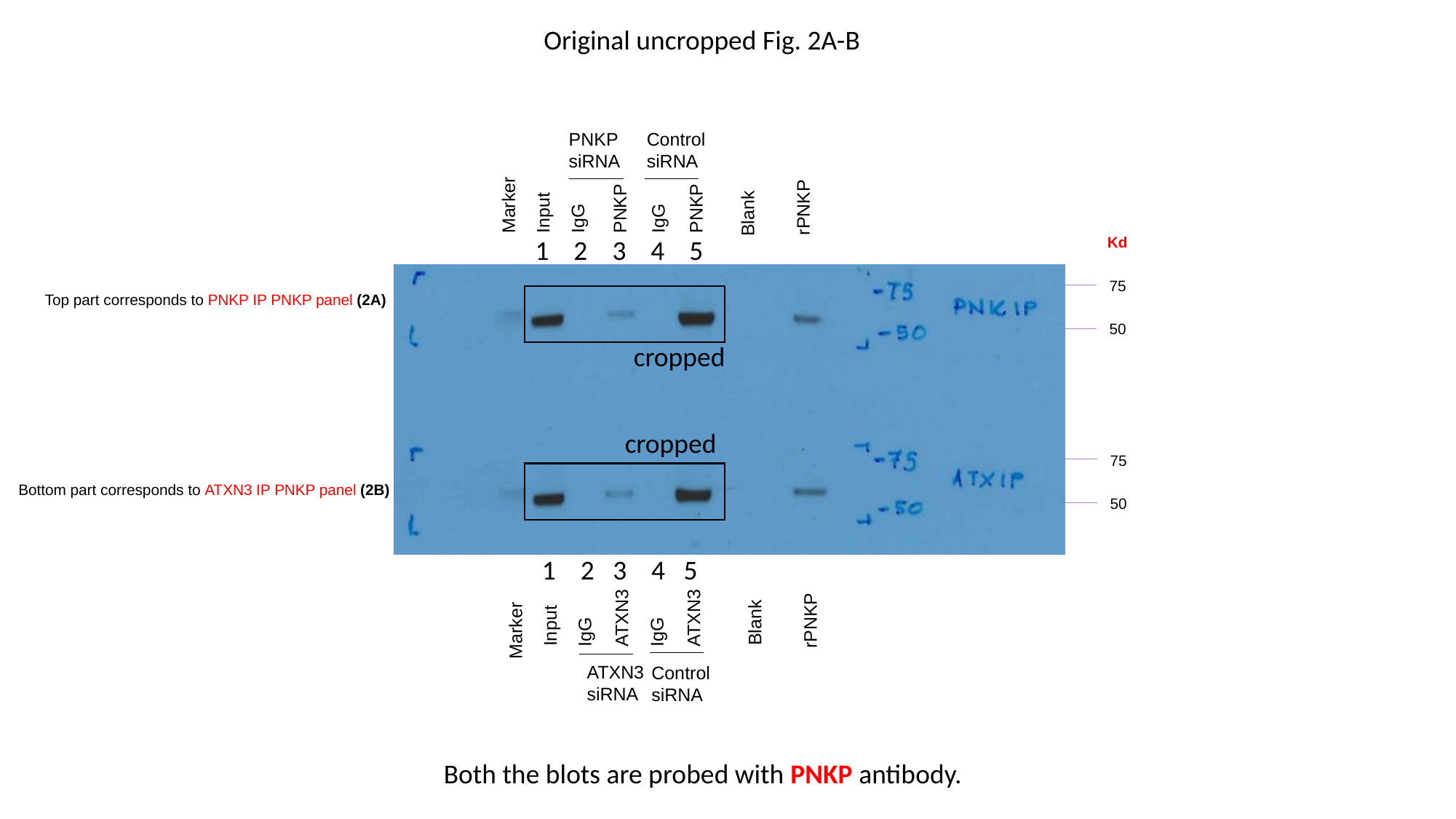

Original uncropped Fig. 2A-B
Control siRNA
PNKP siRNA
Marker
Input
IgG
PNKP
IgG
PNKP
rPNKP
Blank
1 2 3 4 5
Kd
75
Top part corresponds to PNKP IP PNKP panel (2A)
50
cropped
cropped
75
Bottom part corresponds to ATXN3 IP PNKP panel (2B)
50
1 2 3 4 5
Blank
Input
IgG
ATXN3
IgG
ATXN3
rPNKP
Marker
ATXN3 siRNA
Control siRNA
Both the blots are probed with PNKP antibody.

## Slide 3
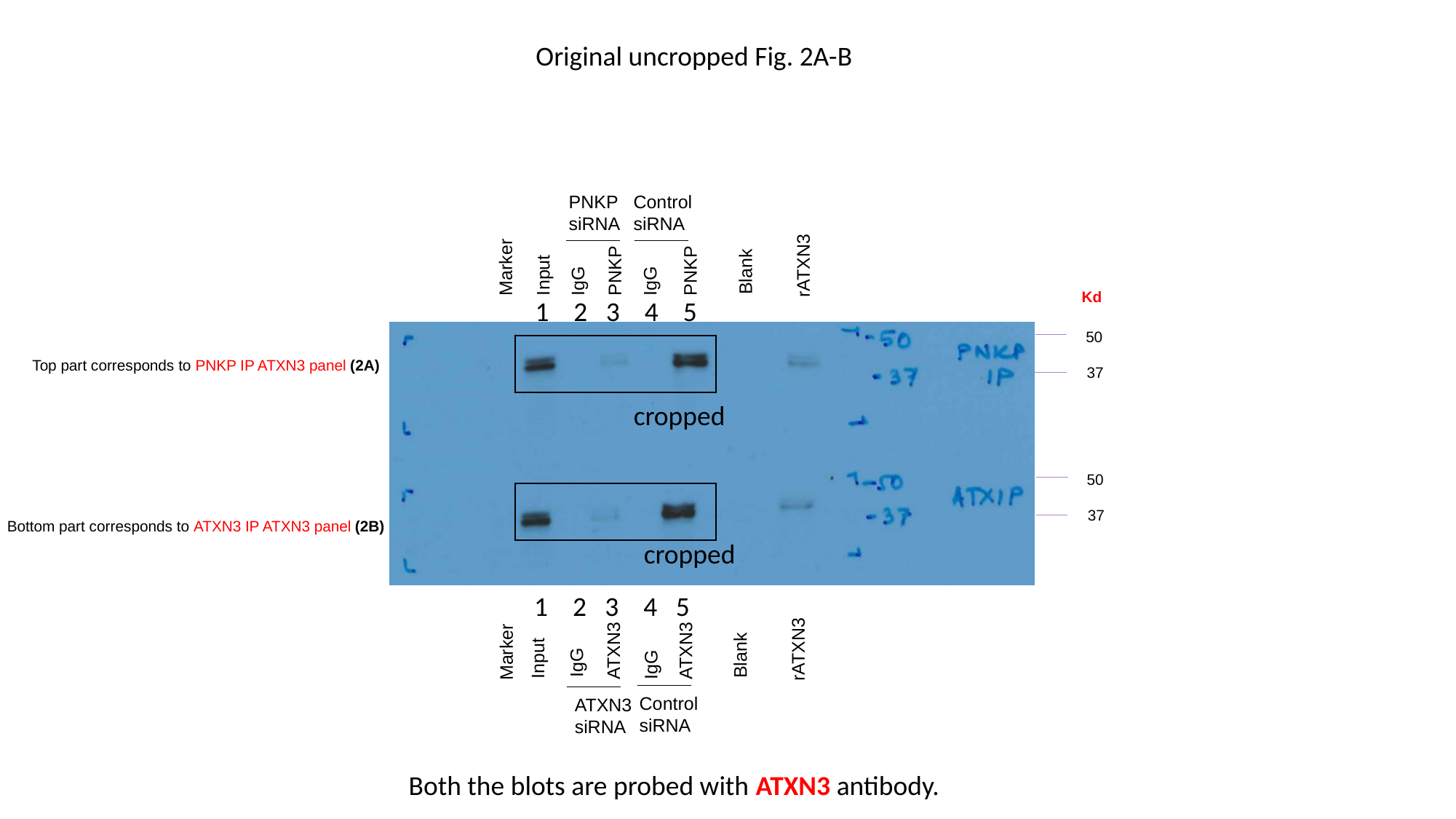

Original uncropped Fig. 2A-B
Control siRNA
PNKP siRNA
Marker
Blank
Input
IgG
PNKP
IgG
PNKP
rATXN3
Kd
1 2 3 4 5
50
Top part corresponds to PNKP IP ATXN3 panel (2A)
37
cropped
50
37
Bottom part corresponds to ATXN3 IP ATXN3 panel (2B)
cropped
1 2 3 4 5
Marker
IgG
Blank
Input
ATXN3
ATXN3
IgG
rATXN3
Control siRNA
ATXN3 siRNA
Both the blots are probed with ATXN3 antibody.

## Slide 4
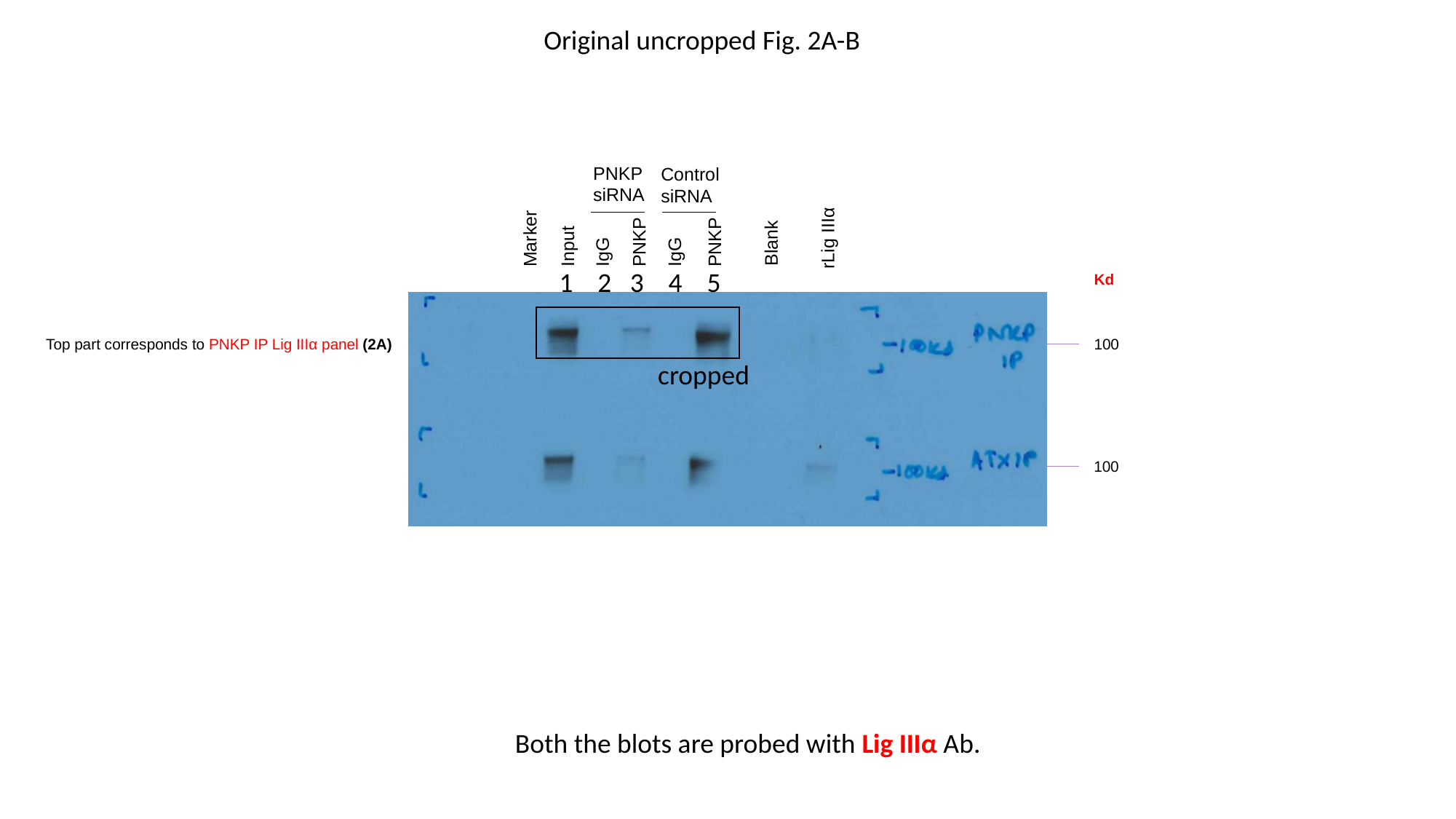

Original uncropped Fig. 2A-B
PNKP siRNA
Control siRNA
Marker
Blank
Input
PNKP
IgG
PNKP
IgG
rLig IIIα
1 2 3 4 5
Kd
Top part corresponds to PNKP IP Lig IIIα panel (2A)
100
cropped
100
Both the blots are probed with Lig IIIα Ab.

## Slide 5
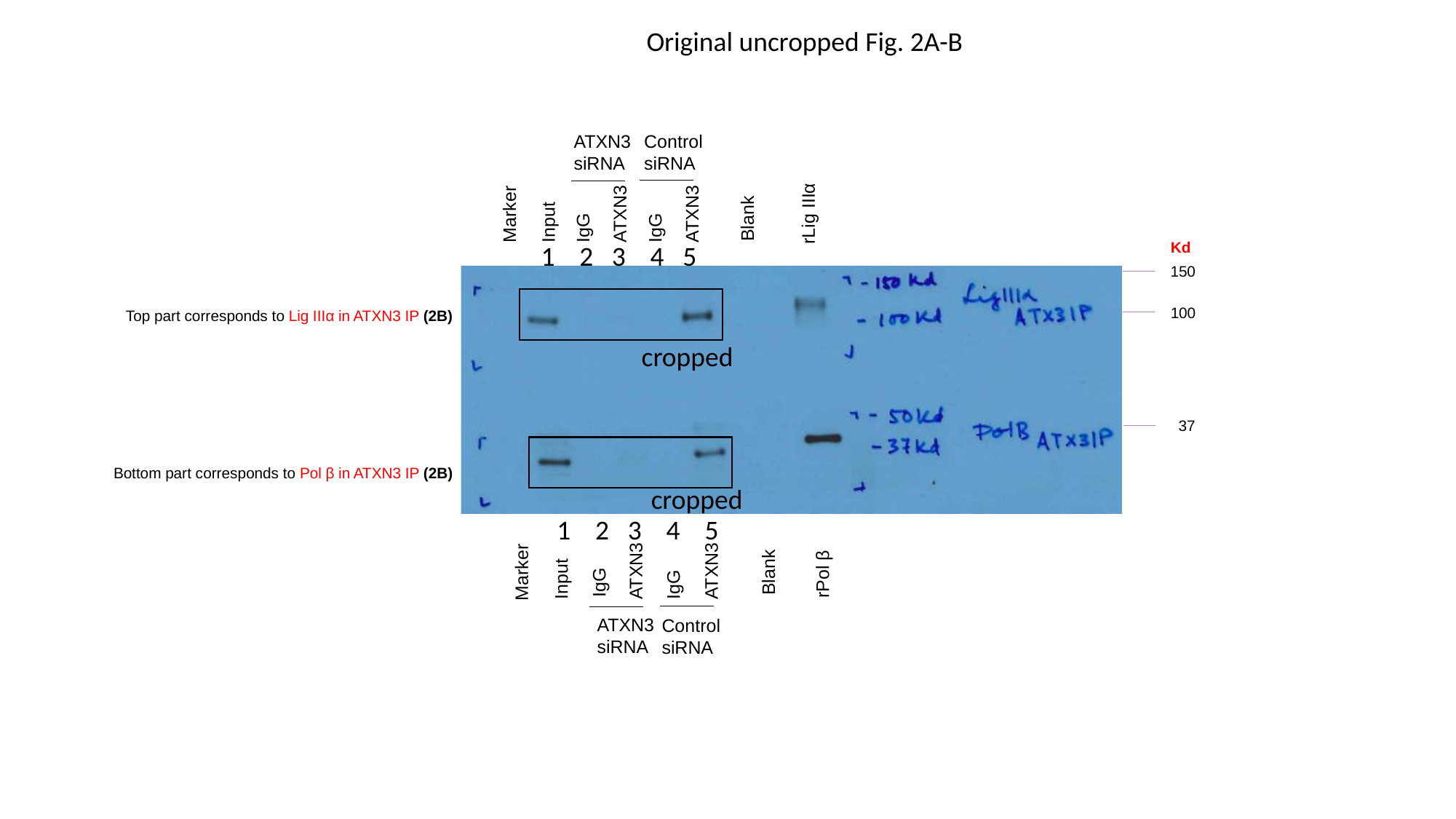

Original uncropped Fig. 2A-B
Control siRNA
ATXN3 siRNA
Marker
Blank
Input
IgG
ATXN3
IgG
ATXN3
rLig IIIα
Kd
1 2 3 4 5
150
100
Top part corresponds to Lig IIIα in ATXN3 IP (2B)
cropped
37
Bottom part corresponds to Pol β in ATXN3 IP (2B)
cropped
1 2 3 4 5
Marker
Blank
IgG
rPol β
Input
ATXN3
ATXN3
IgG
ATXN3 siRNA
Control siRNA

## Slide 6
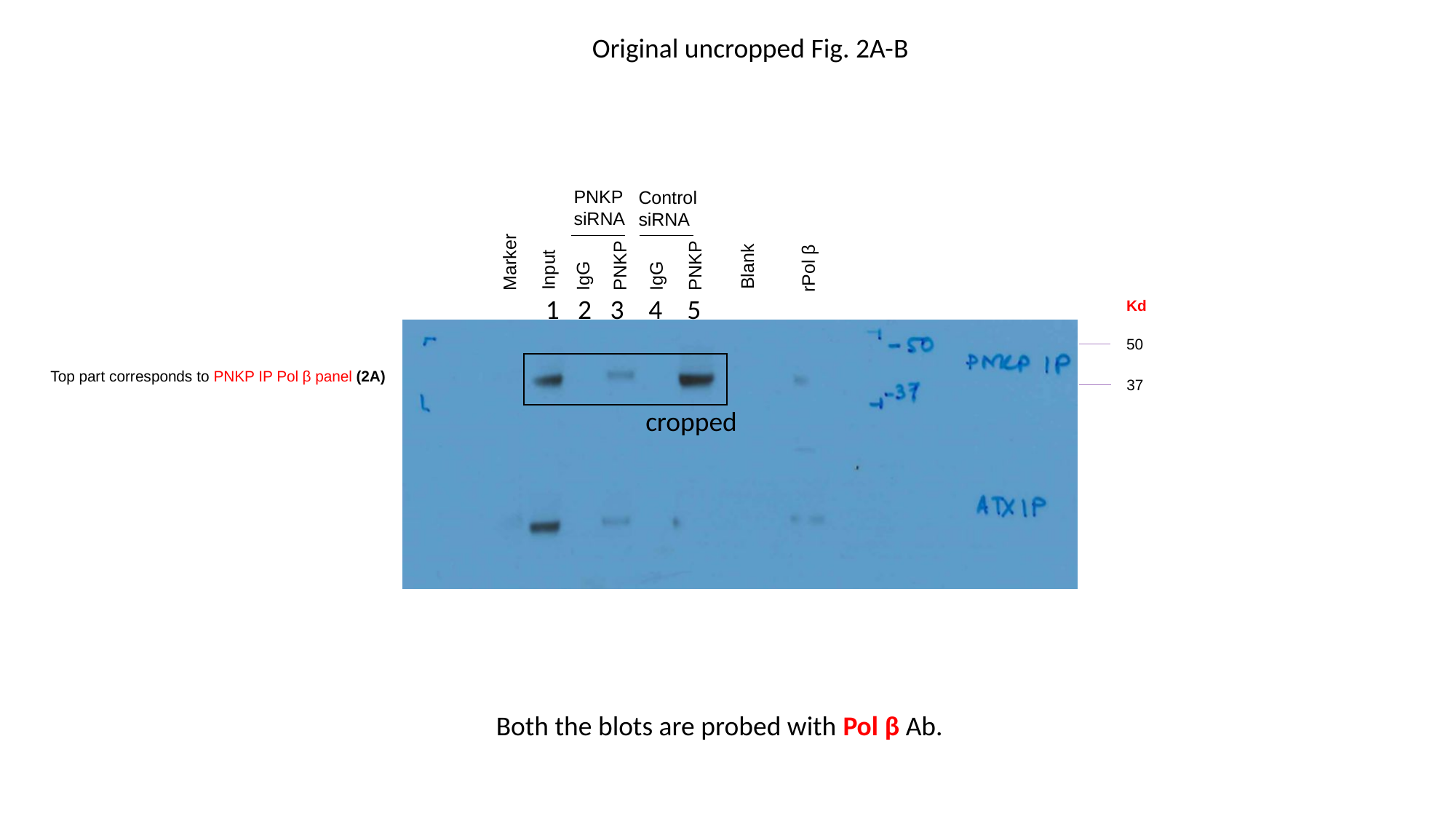

Original uncropped Fig. 2A-B
PNKP siRNA
Control siRNA
Marker
Blank
Input
IgG
PNKP
IgG
PNKP
rPol β
1 2 3 4 5
Kd
50
Top part corresponds to PNKP IP Pol β panel (2A)
37
cropped
Both the blots are probed with Pol β Ab.
