# Supplementary material for: Correction: The Role of the Mammalian DNA End-processing Enzyme Polynucleotide Kinase 3’-Phosphatase in Spinocerebellar Ataxia Type 3 Pathogenesis
Source: PLoS Genet. 2024 Jan 18;20(1):e1011124. doi: 10.1371/journal.pgen.1011124 (PMC10795974; doi:10.1371/journal.pgen.1011124)
Supplement: S2 File — (PPTX) [file pgen.1011124.s002.pptx]

## Slide 1
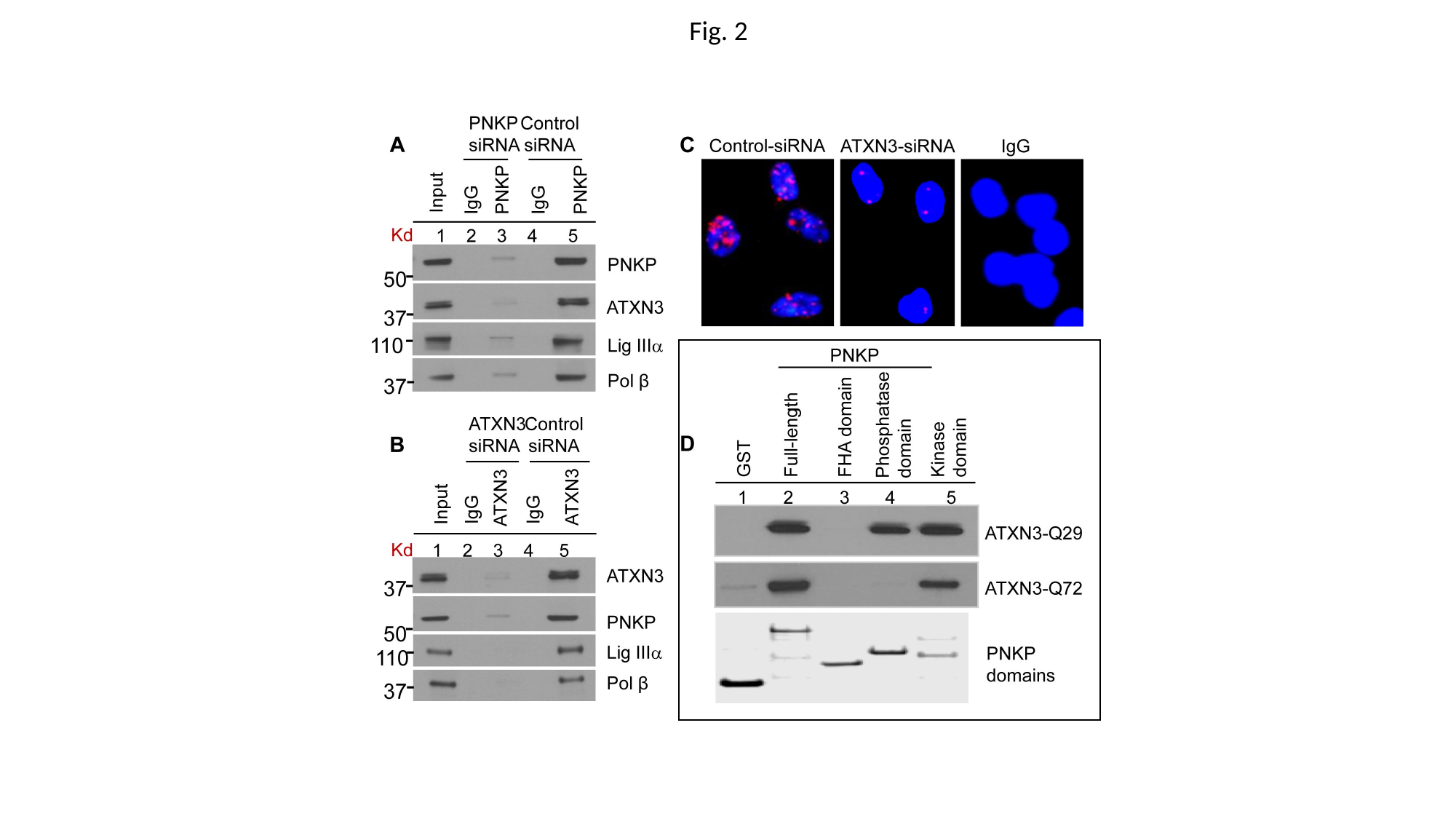

Fig. 2

## Slide 2
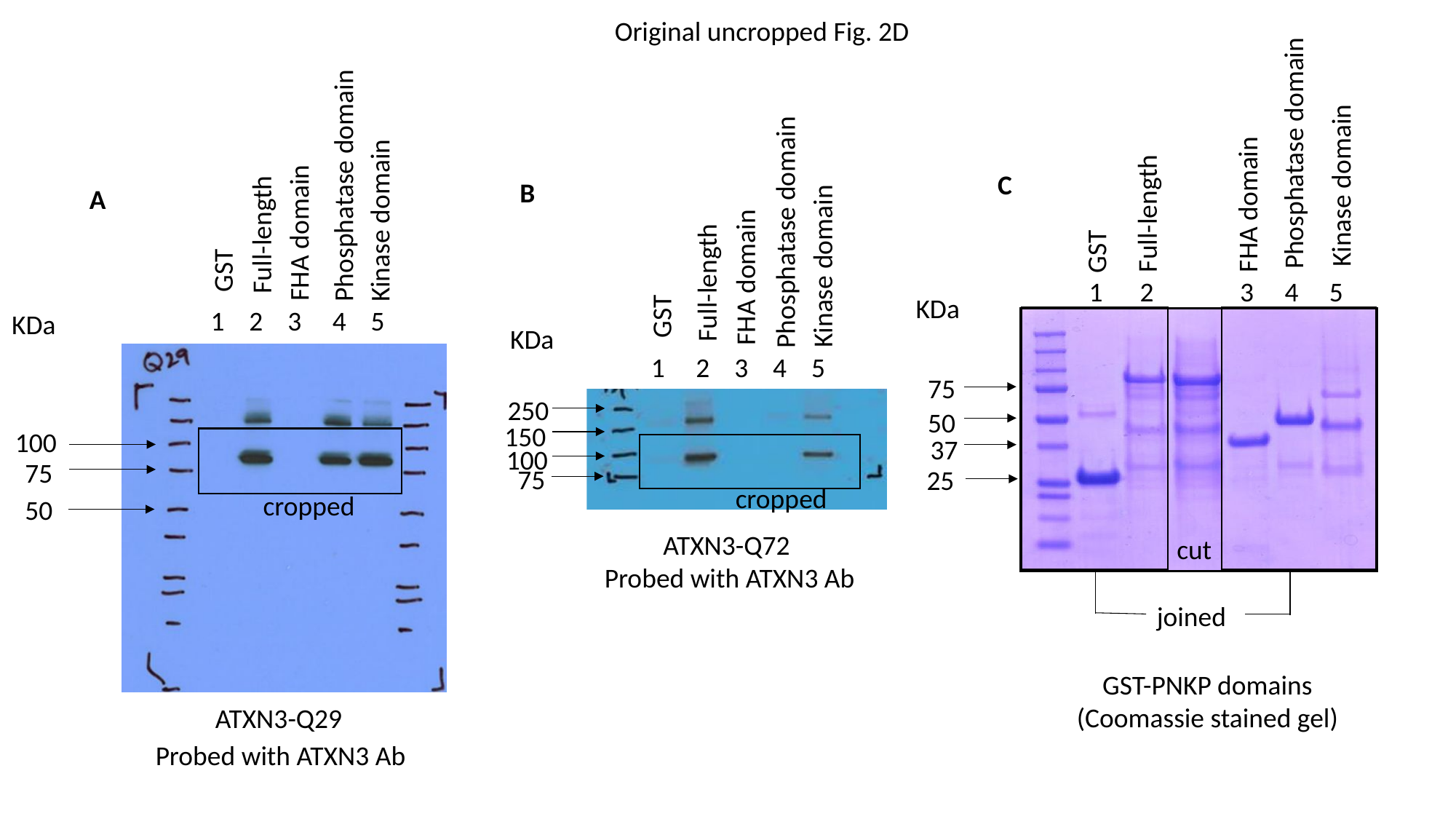

Original uncropped Fig. 2D
Phosphatase domain
Phosphatase domain
Kinase domain
C
B
FHA domain
A
Full-length
Kinase domain
Phosphatase domain
FHA domain
Full-length
GST
Kinase domain
GST
FHA domain
Full-length
 1 2 3 4 5
GST
KDa
1 2 3 4 5
KDa
KDa
1 2 3 4 5
75
250
50
150
100
37
100
75
75
25
cropped
cropped
50
ATXN3-Q72
Probed with ATXN3 Ab
cut
joined
GST-PNKP domains (Coomassie stained gel)
ATXN3-Q29
Probed with ATXN3 Ab
