# Supplementary material for: Correction: The Role of the Mammalian DNA End-processing Enzyme Polynucleotide Kinase 3’-Phosphatase in Spinocerebellar Ataxia Type 3 Pathogenesis
Source: PLoS Genet. 2024 Jan 18;20(1):e1011124. doi: 10.1371/journal.pgen.1011124 (PMC10795974; doi:10.1371/journal.pgen.1011124)
Supplement: S3 File — (PPTX) [file pgen.1011124.s003.pptx]

## Slide 1
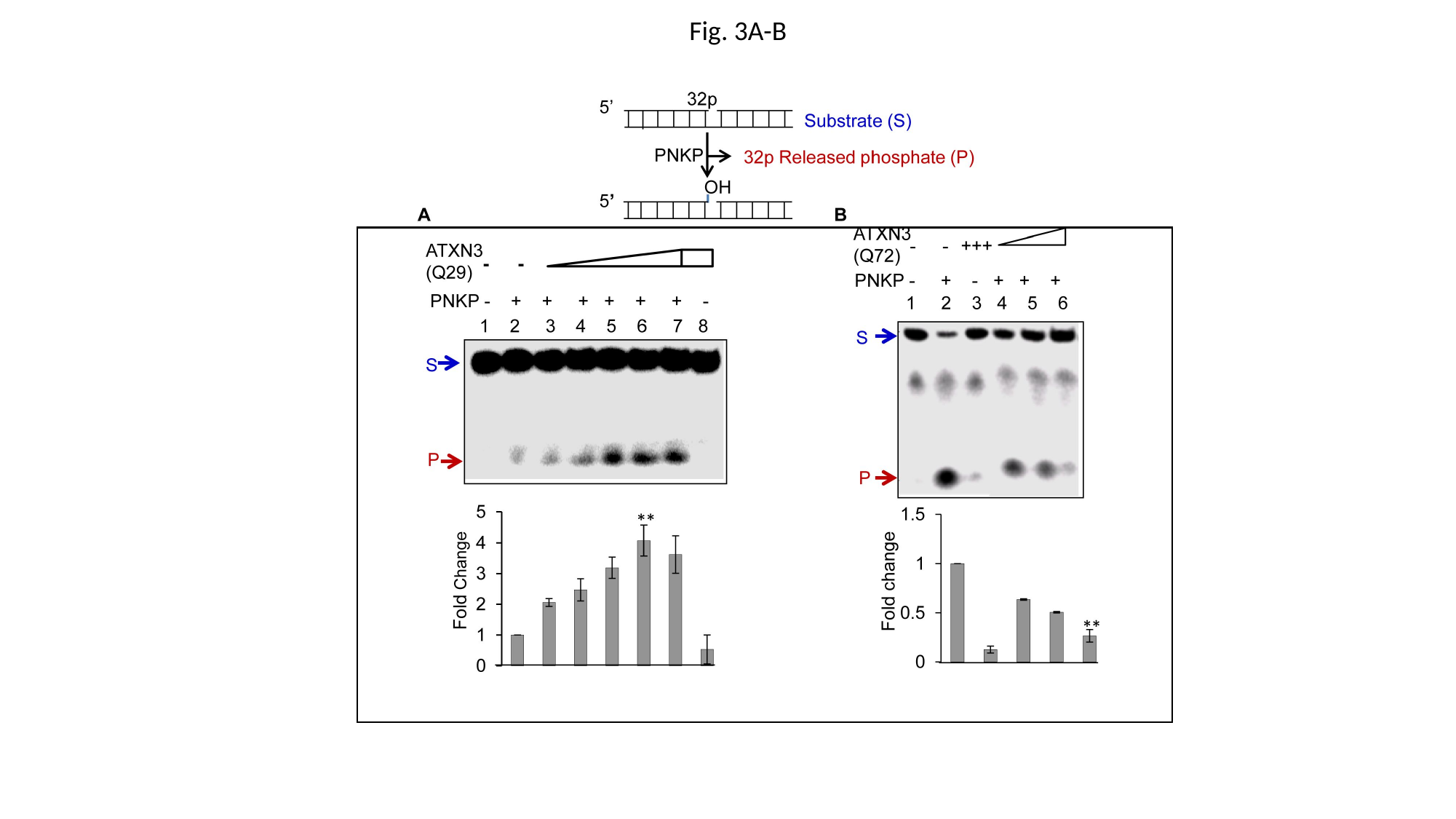

Fig. 3A-B

## Slide 2
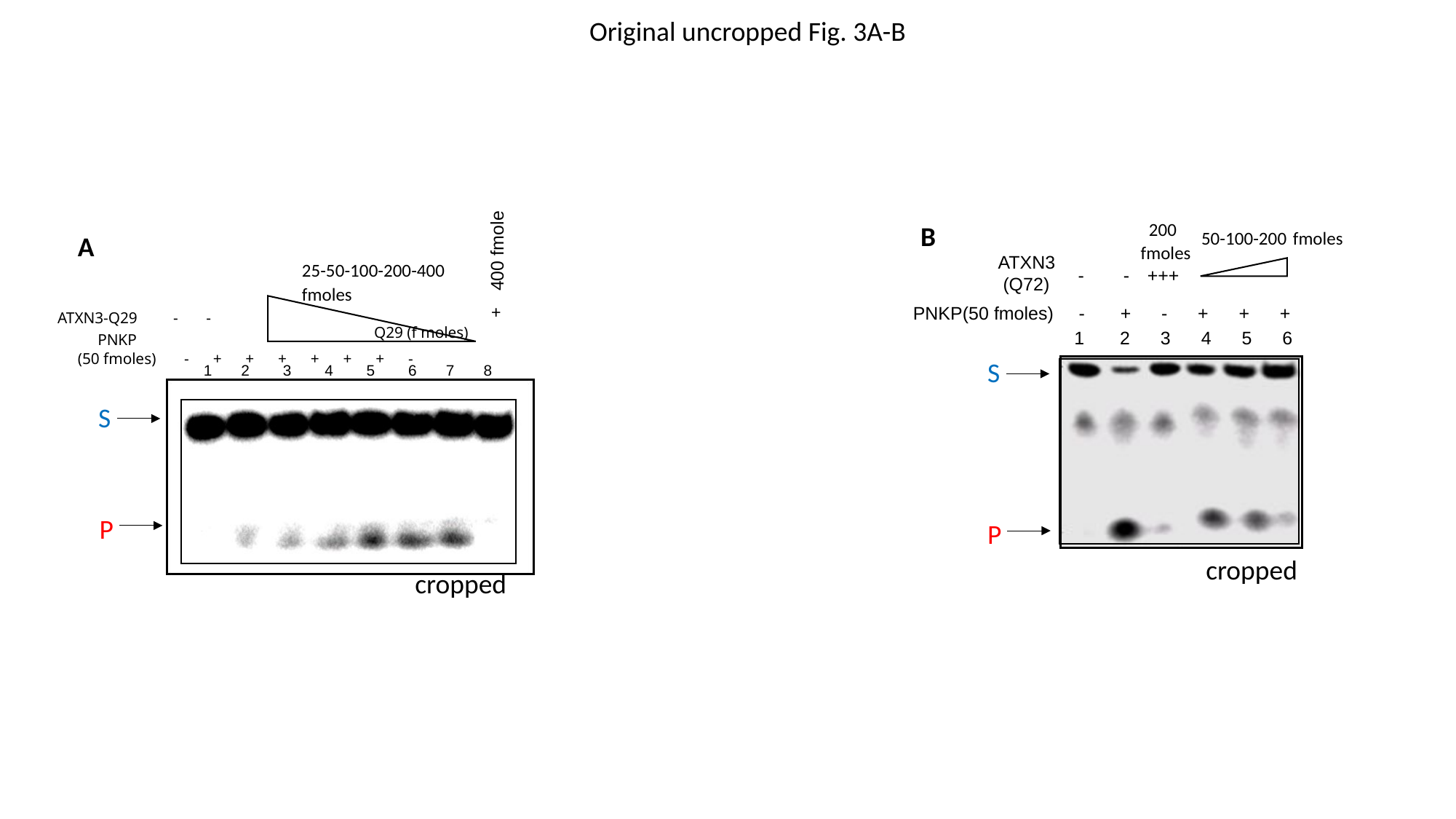

Original uncropped Fig. 3A-B
200
fmoles
50-100-200 fmoles
B
400 fmole
A
25-50-100-200-400 fmoles
ATXN3
 (Q72)
 +++
 -
 -
+
 PNKP(50 fmoles) - + - + + +
 ATXN3-Q29 - -
Q29 (f moles)
 1 2 3 4 5 6
 PNKP
(50 fmoles) - + + + + + + -
S
 1 2 3 4 5 6 7 8
S
P
P
cropped
cropped
