# Supplementary material for: Correction: The Role of the Mammalian DNA End-processing Enzyme Polynucleotide Kinase 3’-Phosphatase in Spinocerebellar Ataxia Type 3 Pathogenesis
Source: PLoS Genet. 2024 Jan 18;20(1):e1011124. doi: 10.1371/journal.pgen.1011124 (PMC10795974; doi:10.1371/journal.pgen.1011124)
Supplement: S4 File — (PPTX) [file pgen.1011124.s004.pptx]

## Slide 1
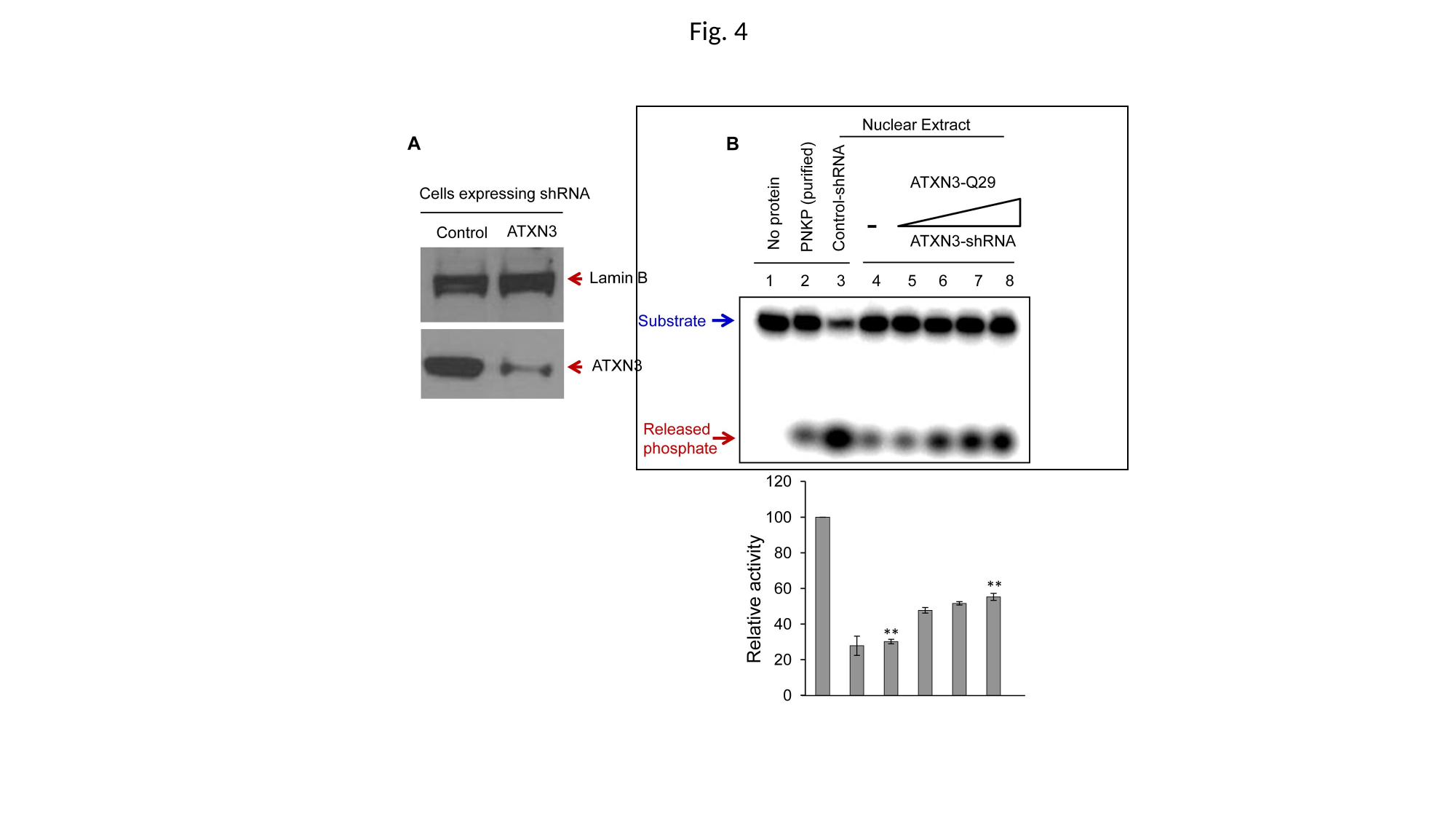

Fig. 4

## Slide 2
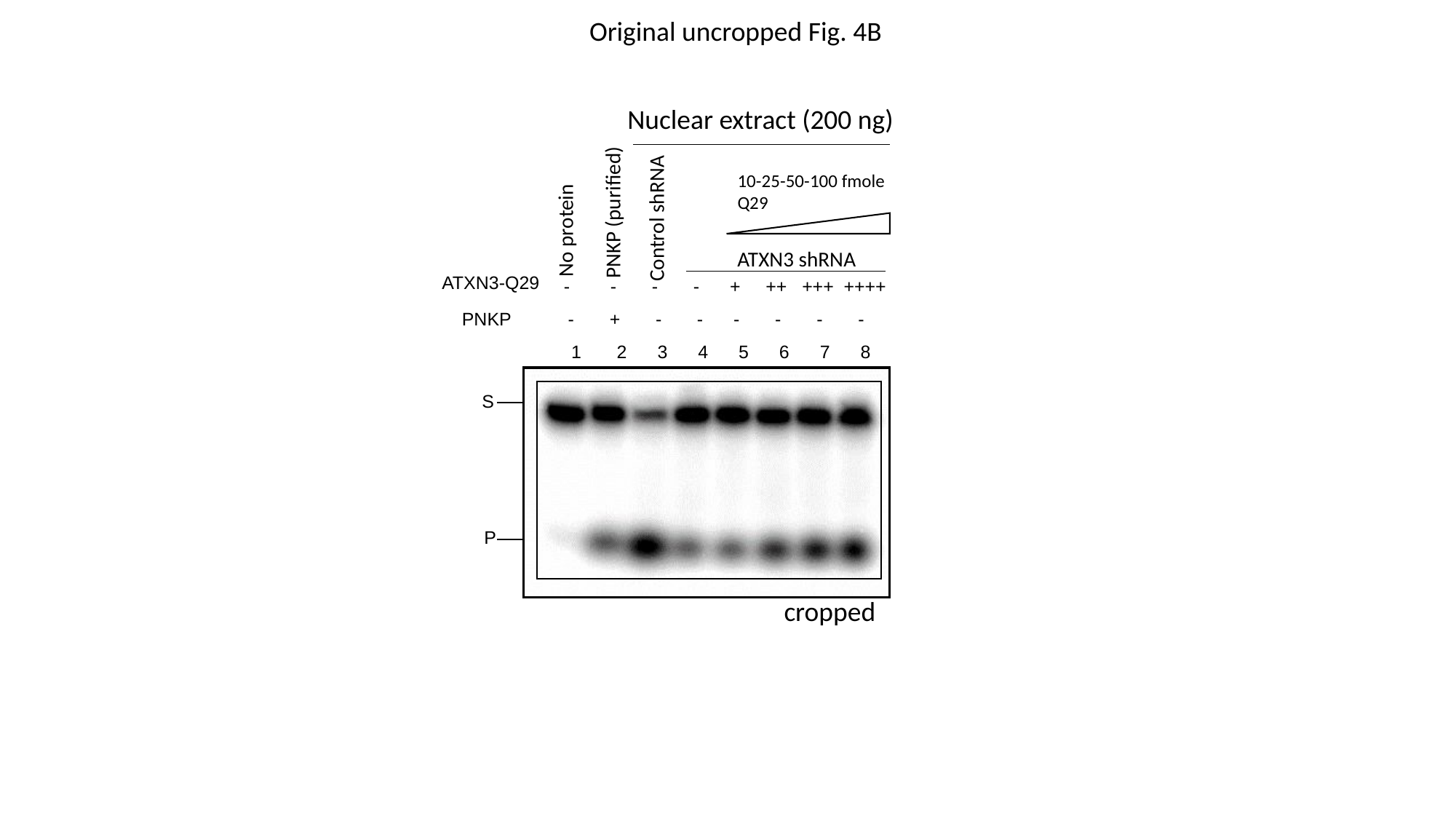

Original uncropped Fig. 4B
Nuclear extract (200 ng)
No protein
10-25-50-100 fmole Q29
PNKP (purified)
Control shRNA
ATXN3 shRNA
ATXN3-Q29
- - - - + ++ +++ ++++
PNKP
 - + - - - - - -
 1 2 3 4 5 6 7 8
S
P
cropped
