# Supplementary material for: Correction: The Role of the Mammalian DNA End-processing Enzyme Polynucleotide Kinase 3’-Phosphatase in Spinocerebellar Ataxia Type 3 Pathogenesis
Source: PLoS Genet. 2024 Jan 18;20(1):e1011124. doi: 10.1371/journal.pgen.1011124 (PMC10795974; doi:10.1371/journal.pgen.1011124)
Supplement: S5 File — (PPTX) [file pgen.1011124.s005.pptx]

## Slide 1
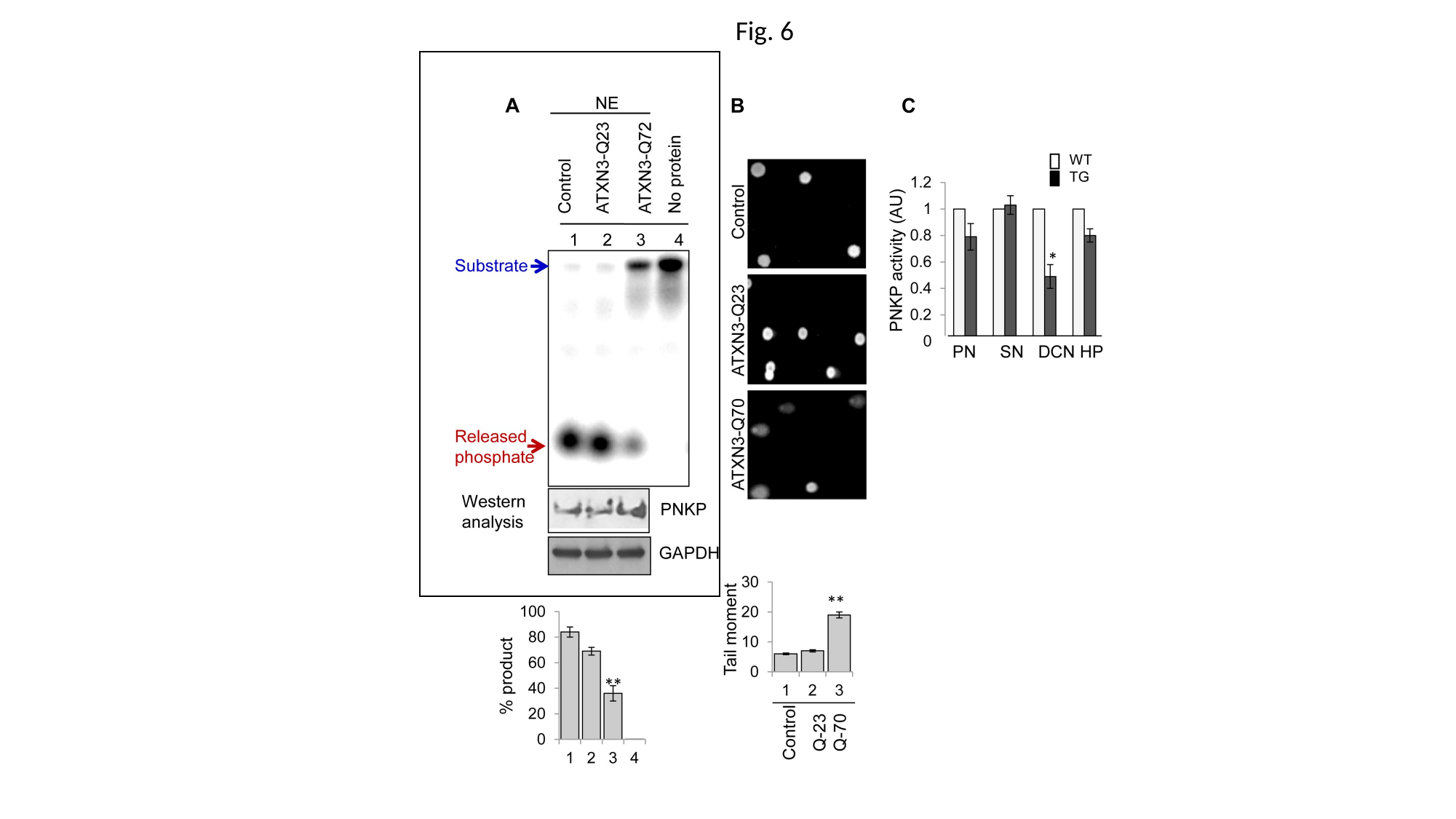

Fig. 6

## Slide 2
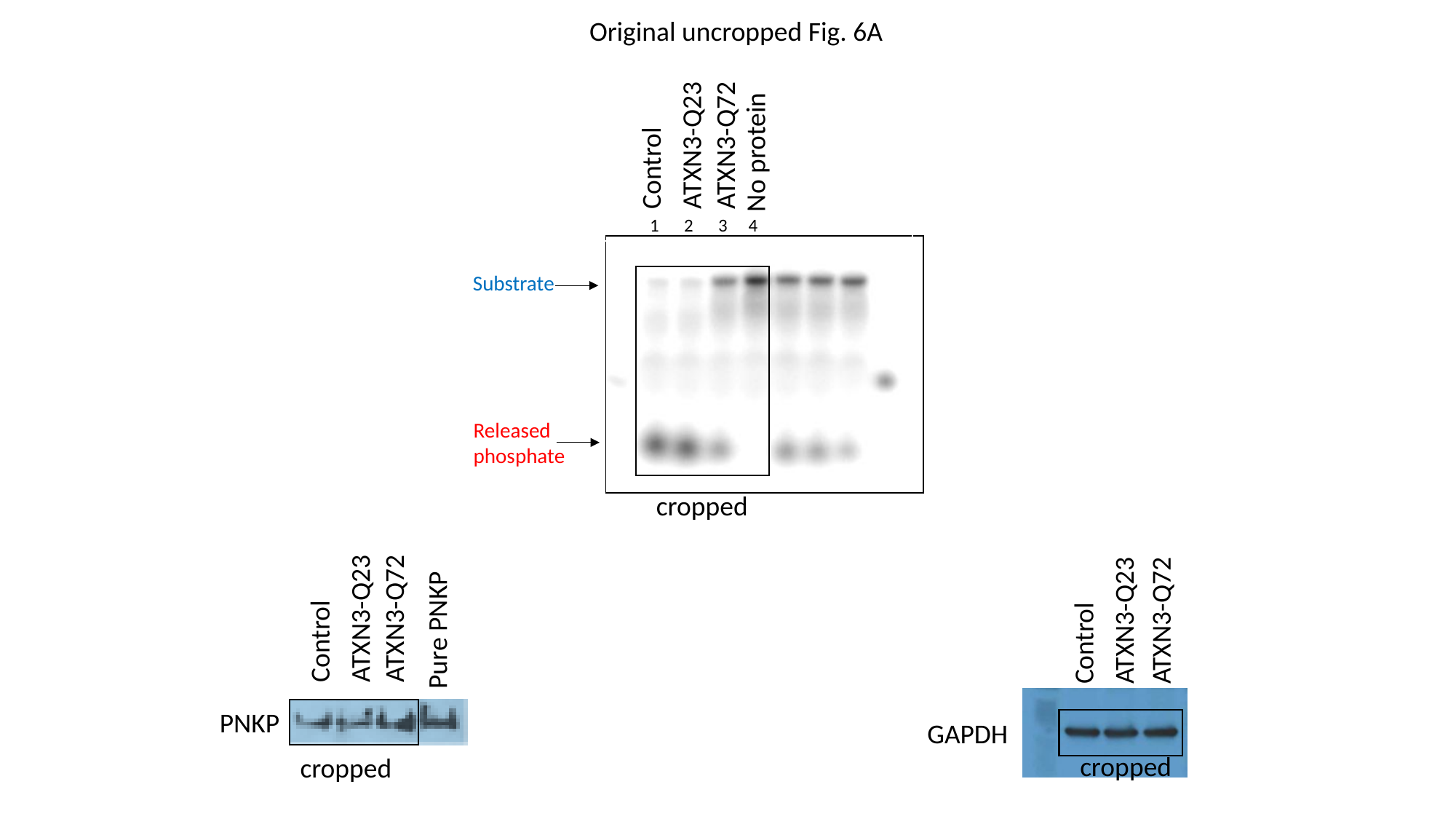

Original uncropped Fig. 6A
ATXN3-Q23
ATXN3-Q72
No protein
Control
 1 2 3 4
Substrate
Released phosphate
cropped
ATXN3-Q23
ATXN3-Q72
ATXN3-Q23
ATXN3-Q72
Pure PNKP
Control
Control
PNKP
GAPDH
cropped
cropped
