# Supplementary material for: Correction: The Role of the Mammalian DNA End-processing Enzyme Polynucleotide Kinase 3’-Phosphatase in Spinocerebellar Ataxia Type 3 Pathogenesis
Source: PLoS Genet. 2024 Jan 18;20(1):e1011124. doi: 10.1371/journal.pgen.1011124 (PMC10795974; doi:10.1371/journal.pgen.1011124)
Supplement: S6 File — (PPTX) [file pgen.1011124.s006.pptx]

## Slide 1
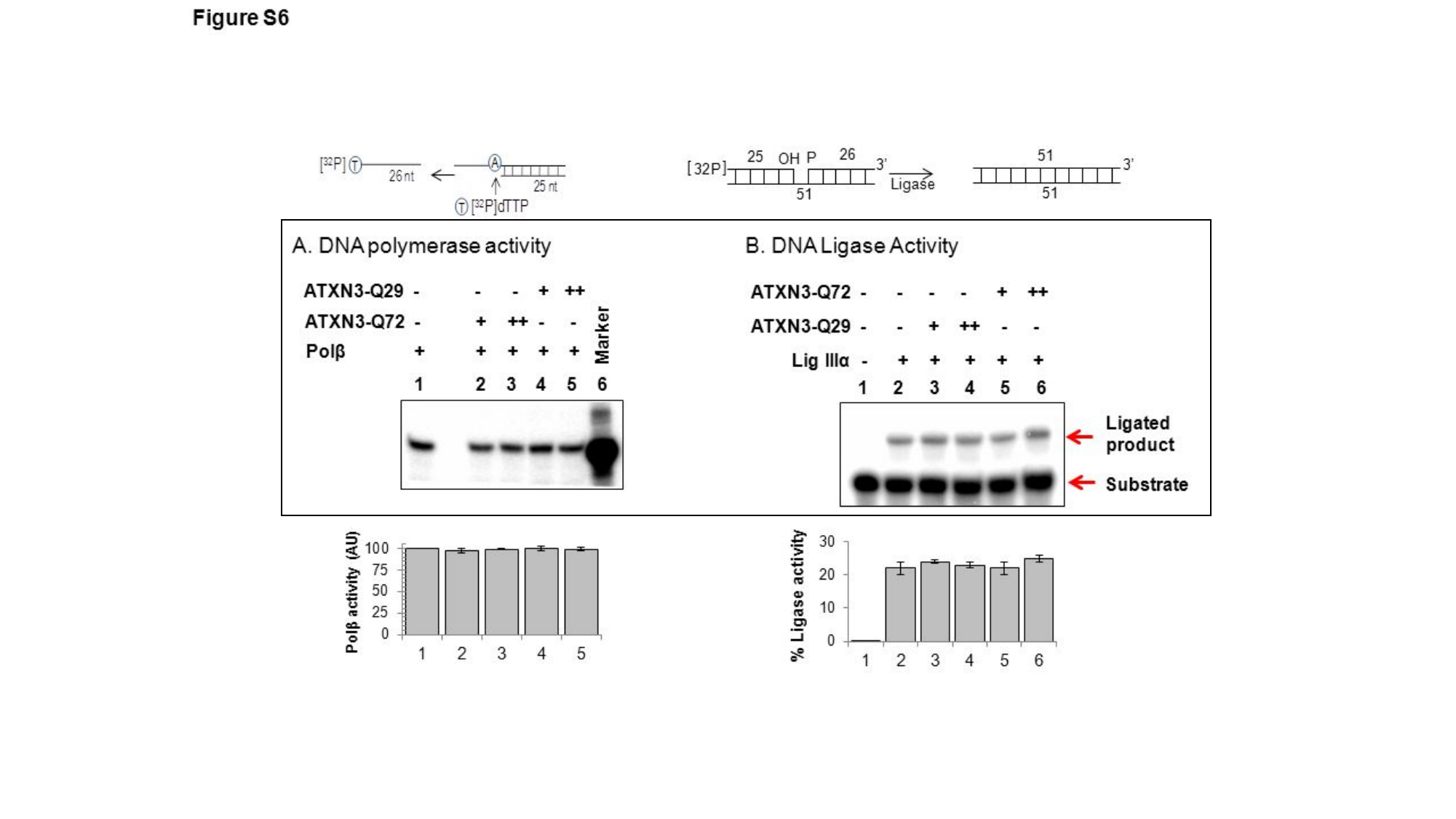

## Slide 2
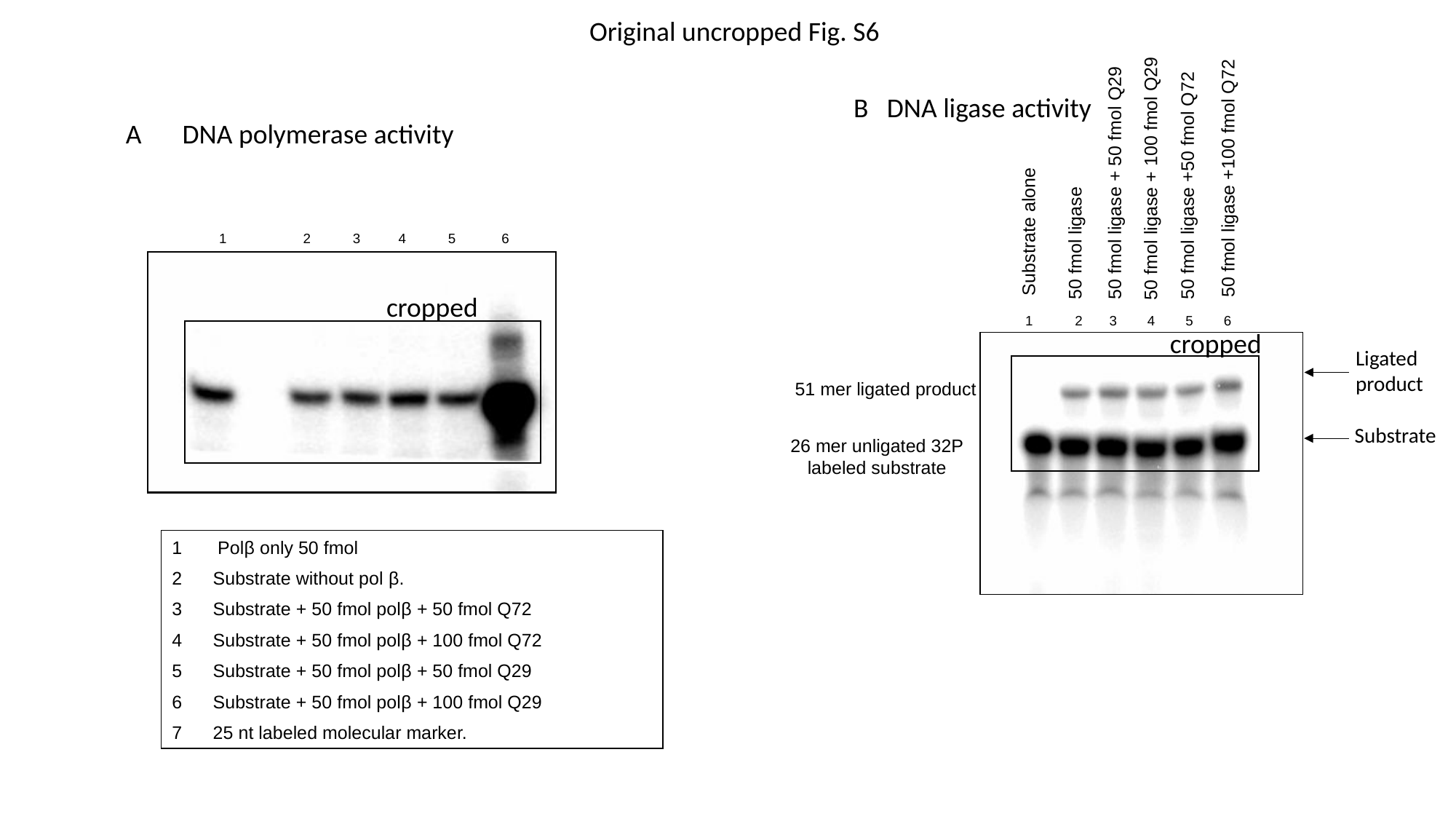

Original uncropped Fig. S6
50 fmol ligase +50 fmol Q72
50 fmol ligase + 50 fmol Q29
50 fmol ligase + 100 fmol Q29
50 fmol ligase +100 fmol Q72
Substrate alone
50 fmol ligase
B
DNA ligase activity
A
DNA polymerase activity
 1 2 3 4 5 6
cropped
 1 2 3 4 5 6
cropped
Ligated product
51 mer ligated product
Substrate
26 mer unligated 32P labeled substrate
1 Polβ only 50 fmol
Substrate without pol β.
Substrate + 50 fmol polβ + 50 fmol Q72
Substrate + 50 fmol polβ + 100 fmol Q72
Substrate + 50 fmol polβ + 50 fmol Q29
Substrate + 50 fmol polβ + 100 fmol Q29
25 nt labeled molecular marker.
